# Potential of ATP5MG to Treat Metabolic Syndrome-Associated Cardiovascular Diseases

**DOI:** 10.3389/fcvm.2022.921778

**Published:** 2022-07-22

**Authors:** Lianyong Liu, Xinglu Zhou, Juan Chen, Xiangqi Li

**Affiliations:** ^1^Department of Endocrinology and Metabolism, Punan Hospital, Shanghai, China; ^2^Department of Endocrinology and Metabolism, Gongli Hospital, Naval Medical University, Shanghai, China; ^3^Department of Obstetrics and Gynecology, Gongli Hospital, Naval Medical University, Shanghai, China

**Keywords:** cardiovascular disease, metabolic syndrome, drug, ATP5L, ceRNA, inflammation, COVID-19, immunity

## Abstract

**Introduction:**

Metabolic syndrome-associated cardiovascular disease (MetS-CVD) is a cluster of metabolism-immunity highly integrated diseases. Emerging evidence hints that mitochondrial energy metabolism may be involved in MetS-CVD development. The physiopathological role of ATP5MG, a subunit of the F0 ATPase complex, has not been fully elucidated.

**Methods:**

In this study, we selected ATP5MG to identify the immunity-mediated pathway and mine drugs targeting this pathway for treating MetS-CVD. Using big data from public databases, we dissected co-expressed RNA (coRNA), competing endogenous RNA (ceRNA), and interacting RNA (interRNA) genes for ATP5MG.

**Results:**

It was identified that ATP5MG may form ceRNA with COX5A through hsa-miR-142-5p and interplay with NDUFB8, SOD1, and MDH2 through RNA–RNA interaction under the immune pathway. We dug out 251 chemicals that may target this network and identified some of them as clinical drugs. We proposed five medicines for treating MetS-CVD. Interestingly, six drugs are being tested to treat COVID-19, which unexpectedly offers a new potential host-targeting antiviral strategy.

**Conclusion:**

Collectively, we revealed the potential significance of the ATP5MG-centered network for developing drugs to treat MetS-CVD, which offers insights into the epigenetic regulation for metabolism-immunity highly integrated diseases.

## Introduction

Metabolic syndrome-associated cardiovascular disease (MetS-CVD) represents a cluster of metabolic deformities that contribute to increased oxidative stress and activated inflammatory pathways that cause cardiovascular remodeling and dysfunction (1). This constellation of biochemical, molecular, and clinical abnormalities is related to diabetes mellitus and obesity, eventually cardiovascular events and death (2). Just as is known, metabolic regulation and immune-inflammatory response are highly integrated and interdependent, and the deregulation of this central homeostatic mechanism can cause a cluster of diseases, such as MetS, obesity, diabetes, and CVD (3). These factors hint that the immunity/inflammation-related pathway may be a critical pathological mediator underlying MetS-CVD. However, therapeutic strategies targeting this signaling pathway to prevent and/or treat MetS-CVDs remain limited.

Emerging evidence proposes that energy metabolism may be involved in developing MetS-CVD besides substance metabolisms such as glucose and lipid dysregulation (4, 5). Moreover, relatively less data are expected to reinforce this line of research. As its name suggests, ATP5MG/ATP5L, a subunit of the F0 ATPase complex, may function in mitochondrial energy metabolism. These studies do not fully elucidate its function. To our relief, there are still some functional clues, which are primarily from differential expression analysis. ATP5L expression can be regulated by shear stress in human coronary artery endothelial cells (6). ATP5L deregulation is related to the dynamic transition from obesity to T2D (7) and the development of hypertension (8). MiR-570-3p can regulate ATP5L with concomitant ATP loss in platelets (9). ATP5L dysfunction impairs energy metabolism (10) and causes reduced cerebrospinal fluid (CSF) production in Alzheimer's disease (AD) (11). ATP5L is possibly involved in acute mountain sickness (12) and PM2.5 exposure-related lung pathogenesis (13). ATP5L may act as a marker of oncogenic cell transformation (14) and be upregulated using orchiectomy in the substantia nigra in aged males (15). Additionally, ATP5L-KMT2A gene fusion presents in leukemia (16, 17). It can be seen from the above examinations that the association between ATP5MG and MetS-CVD development has not been shown. ATP5MG involvement in inflammatory/immune pathways to influence the pathology of MetS-CVD remains to be identified.

MetS-CVD has a systemic effect, by intersecting ceRNA (competing endogenous RNA) genes and interRNA (interacting RNA) genes in the whole body with genes co-expressed in the heart, we looked for the ATP5MG-circled network for energy metabolic effects on the heart dysfunction using the inflammatory or immune pathway. We further identified chemicals perturbing this network to dig medicines for treating MetS-CVD. Our study may provide epigenetic insights into metabolism-immunity/inflammation highly integrated diseases for basic research and drug development.

## Materials and Methods

Free public resources, including tools and databases, were enlisted to obtain data and draw figures. To guarantee the objectivity of the result, all analyses throughout the study were employed according to the default settings of the database or tool, unless otherwise stated. When conducting analysis using default parameters, these excellent foolproof operating tools or databases displayed needed data instantly for free download. Figures were made using POWERPOINT or EXCEL unless otherwise specified.

The online tool DISEASES (18) was used to analyze the associations of ATP5MG with diseases. Only the human gene ATP5MG was selected, and the Z-score data were downloaded by selecting “text mining.” All genes of ceRNA and interRNA of ATP5MG were obtained from ENCORI (19) for functional analysis. For ceRNA, the parameters are as follows: miRNA number ≥2, *P*-value, and false discovery rate (FDR) both ≤ 0.01; for interRNA, interact number ≥1, experiment number ≥1. GTEx data in the GEPIA2 database (20) was employed to extract two hundred coRNA genes of ATP5MG in the heart by conducting the Pearson's correlation coefficient (PCC) analysis. For overlapping genes between coRNA and ceRNA or interRNA, their correlations with ATP5MG were presented online and downloaded as scatter plots and *p*-values and correlation coefficients. Gene function comments including GOTERM-BP, GOTERM-CC, GOTERM-MF, Kyoto Encyclopedia of Genes and Genomes (KEGG) PATHWAY, HIV INTERACTION (CATEGORY), and GAD DISEASE (CLASS) for coRNA, ceRNA, and interRNA datasets were achieved using well-known tool DAVID (21) and illustrated using the glistening tool IMAGEGP.

Another eyeable tool, EVENN, was used to present intersection genes (22). TissueAtlas was invited to profile miRNA expression in distinct tissues and present the network of miRNA in the heart (23, 24). The related miRNA expression data were obtained from Next Generation Sequencing (NGS) and normalized using RPMM. Functional information about all intersection genes was downloaded from InnatedDB (25). Only human gene was selected, and Gene Ontology (GO) terms were extracted directly. KEGG analysis for ATP5MG and four intersection genes between coRNA and ceRNA genes was conducted using KOBAS (26). The statistical method used was “hypergeometric test/Fisher's exact test,” and the FDR correction method was “Benjamini and Hochberg.” Tissue and cell profiling data of intersection genes were directly downloaded from HPA, a brilliant online database set including massive amounts of human RNA and protein expression information (27). Functional processes of intersection genes were downloaded from NCBI and drawn using IMAGEGP.

Gene–chemical interactions were queried using the gene symbol from the Comparative Toxicogenomics Database (CTD) database, a repository embodying the effects of environmental chemicals on human gene expression (28). All the compounds that hit the targets, ATP5MG, COX5A, NDUFB8, SOD1, and MDH2, were downloaded. The network of gene–chemical interplay was visualized using the software Cytoscape (29), that is, dragging text file including genes and chemicals directly to the window of the network and the targeted network is automatically visualized. Drug matching analysis was performed using DrugBank to dissect potential drug effects for all these chemicals, and clinical trial data for queried chemicals in DrugBank were downloaded and manually performed disease classification analysis one by one (30).

## Results

### Deploy MetS-CVD as the Target of the Analysis of ATP5MG

**Figure 1:**
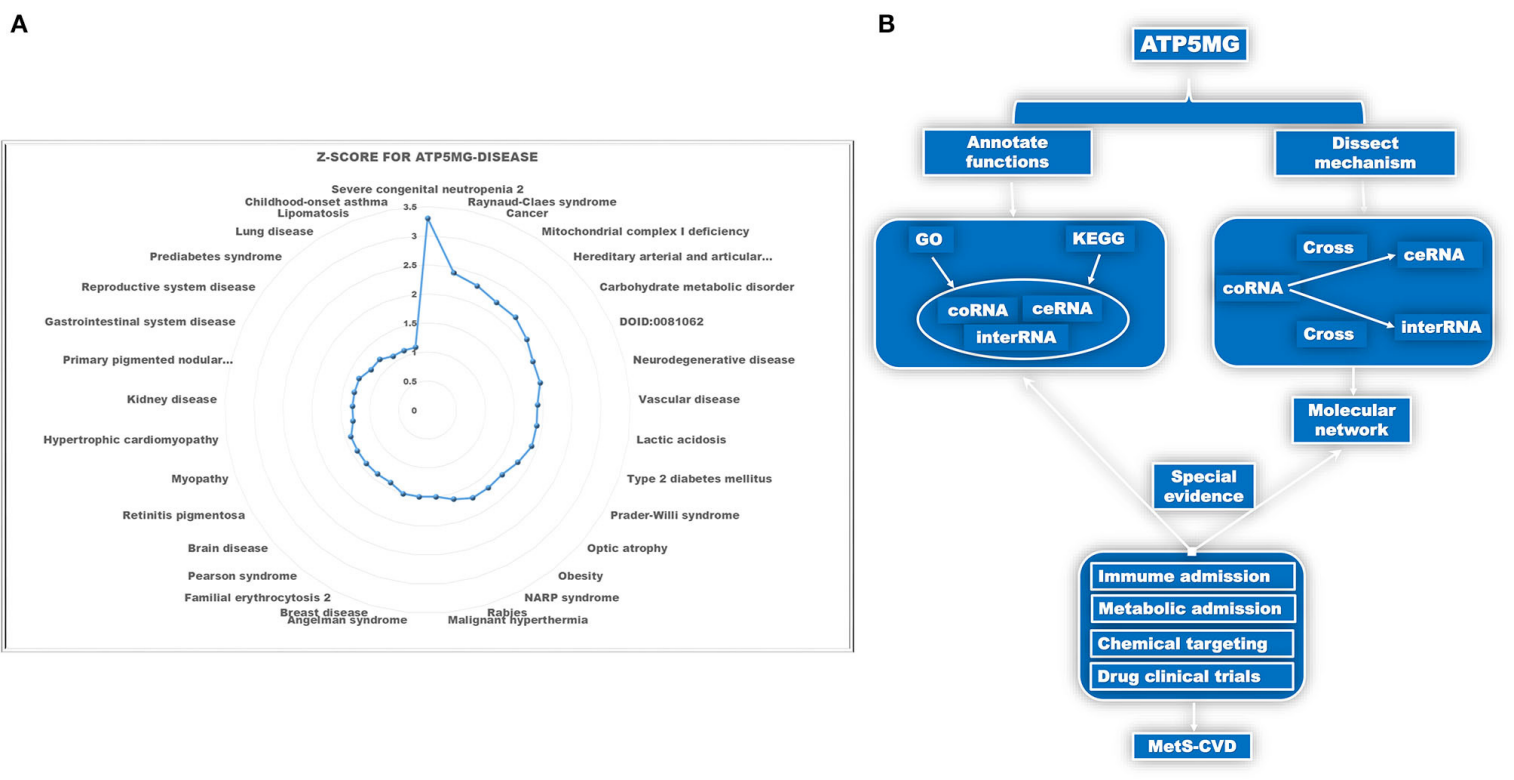
Schematic diagram of the project design. **(A)** Dissect ATP5MG–disease associations by Z-score. **(B)** Design analysis scheme for biological functions and the underlying mechanisms of ATP5MG in MetS-CVD.

Identifying the involvement of the ATP5MG-mediated immune-inflammatory pathway in MetS-CVD is not easy. As MetS-CVD is a complex systemic disease and often related to several diseases such as diabetes, obesity, and heart disease, we enlisted the tool DISEASES to parse ATP5MG–disease associations mined from the literature. Only 33 types of disease associations were uncovered (Figure 1A), and “severe congenital neutropenia 2,” “mitochondrial disease,” “vascular disease,” “type 2 diabetes mellitus,” “obesity,” “hypertrophic cardiomyopathy,” and “prediabetes syndrome” were included (Figure 1A). This means that ATP5MG may be involved in immune and metabolic diseases. The highest *Z*-score was only 3.3 for “severe congenital neutropenia 2.” Considering that the number of disease associations was small and the highest score was low, it was concluded that the functional ATP5MG studies were presently weak, which supported the high originality of our project design.

The pathogenesis of complex diseases must involve complex molecular networks. Hence, to determine the functional annotations and molecular mechanisms underlying MetS-CVD, we used the coRNA, ceRNA, and interRNA genes of ATP5MG to conduct all the analyses. GO and KEGG were enlisted to annotate their functions. Intersections of coRNA and ceRNA genes and coRNA and interRNA genes were used to obtain molecules to form our target network (Figure 1B). As a big data article, to verify our molecular network, we selected a highly efficient approach that is distinct from traditional experimental and bioinformatics ones. We used the CTD database to search for environmental chemicals that can act on our network and looked for clinical drugs from these chemicals that may treat diabetes, heart disease, or immunity/inflammation-mediated diseases (Figure 1B). The existence of such drugs is equivalent to providing strong evidence of the reasonableness of our molecular network.

### Biological Roles of ATP5MG in the Human Heart by CoRNA Analysis

Due to less verified functional information on ATP5MG, we first set out to prospect its functional notes in the heart. We extracted 200 coRNA genes in the human heart from GEPIA2 and dissected functional annotations of ATP5MG using the tool DAVID.

**Figure 2:**
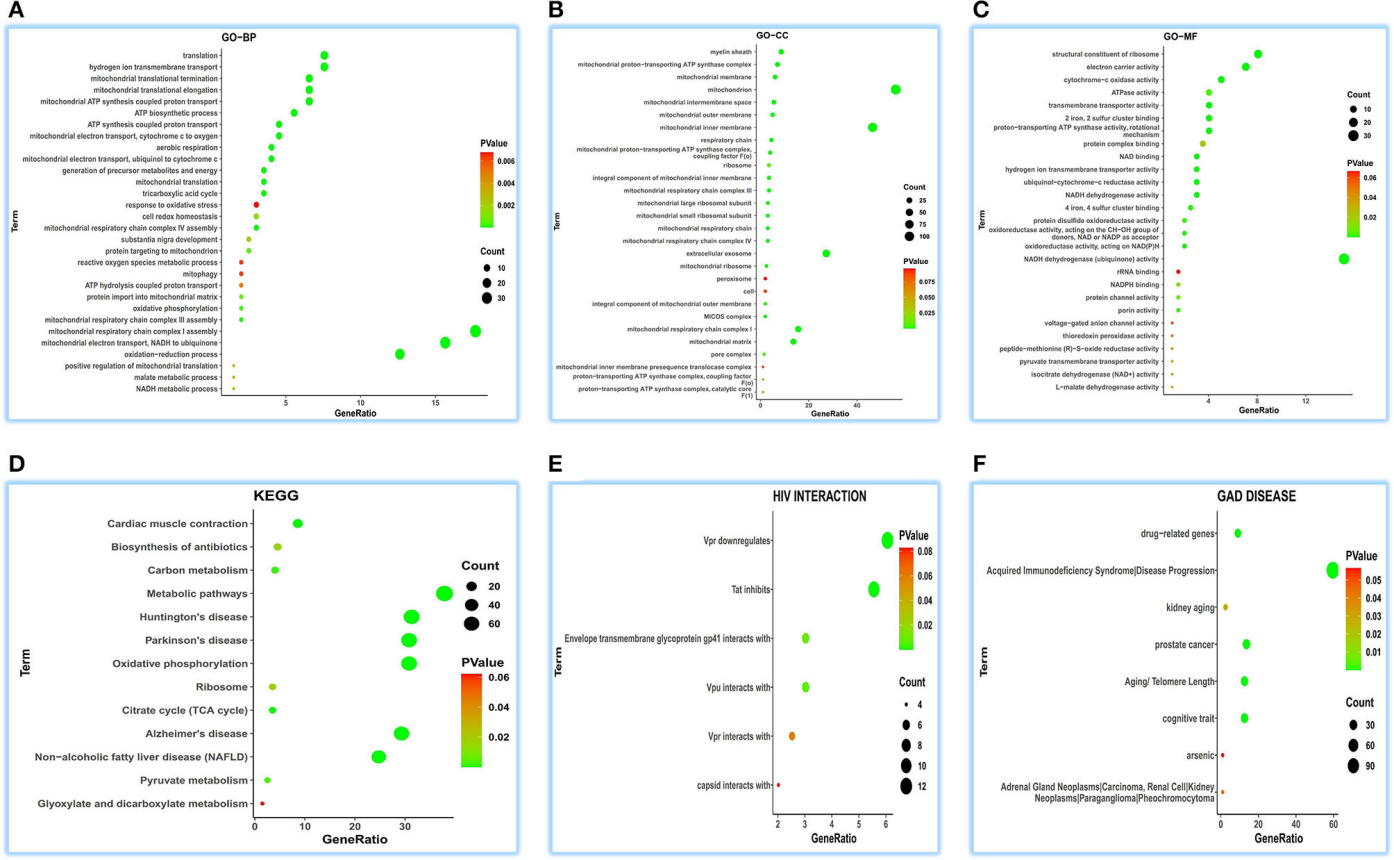
Functional annotations of ATP5MG in the human heart by coRNA analysis. The analysis of GO, KEGG, HIV interaction and GAD DISEASE based on two hundreds of coexpression genes of ATP5MG was conducted using DAVID. ATP5MG may present various functions, obviously focused on mitochondrial energy metabolism and related to infection, inflammation, or immune-related function in the heart. **(A)** Functional annotations by GO-BP analysis. **(B)** Functional annotations by GO-CC analysis. **(C)** Functional annotations by GO-MF analysis. **(D)** Functional annotations using KEGG analysis. **(E)** Functional annotations by HIV interaction analysis. **(F)** Functional annotations by GAD DISEASE analysis. GO, Gene Ontology; BP, biological process; CC, cellular component; MF, molecular function. KEGG, Kyoto Encyclopedia of Genes and Genomes.

Concerning GO-BP, we visualized the top 30 terms in 48 terms (Figure 2A) and found that its function was enriched in mitochondrial energy metabolism besides some metabolic and redox terms. Regarding GO-CC, we got 28 terms, showing a focused expression in mitochondria (Figure 2B). For GO-MF, we obtained 27 terms; still, involving energy metabolism was a focus (Figure 2C). KEGG pathway analysis indicates its wide involvement in the disease process in 13 terms, such as “oxidative phosphorylation,” “non-alcoholic fatty liver disease (NAFLD),” “metabolic pathways,” “cardiac muscle contraction,” and “biosynthesis of antibiotics” (Figure 2D). Furthermore, GAD DISEASE analysis for eight terms disclosed its role in acquired immunodeficiency syndrome, aging, and cancer (Figure 2E). HIV interaction analysis for six terms (Figure 2F) presented its role in viral infections.

As observed in the above coRNA analysis, the ATP5MG functions are broad, including energy regulation, inflammation/infection, and substance metabolism, of which energy production is a high-frequency term.

### Comprehensive Functions of ATP5MG by CeRNA Analysis

A total of 1,326 ceRNA genes extracted from ENCORI were parsed for functional comments in human tissues using DAVID. The top 30 terms for all the sets were visualized.

**Figure 3:**
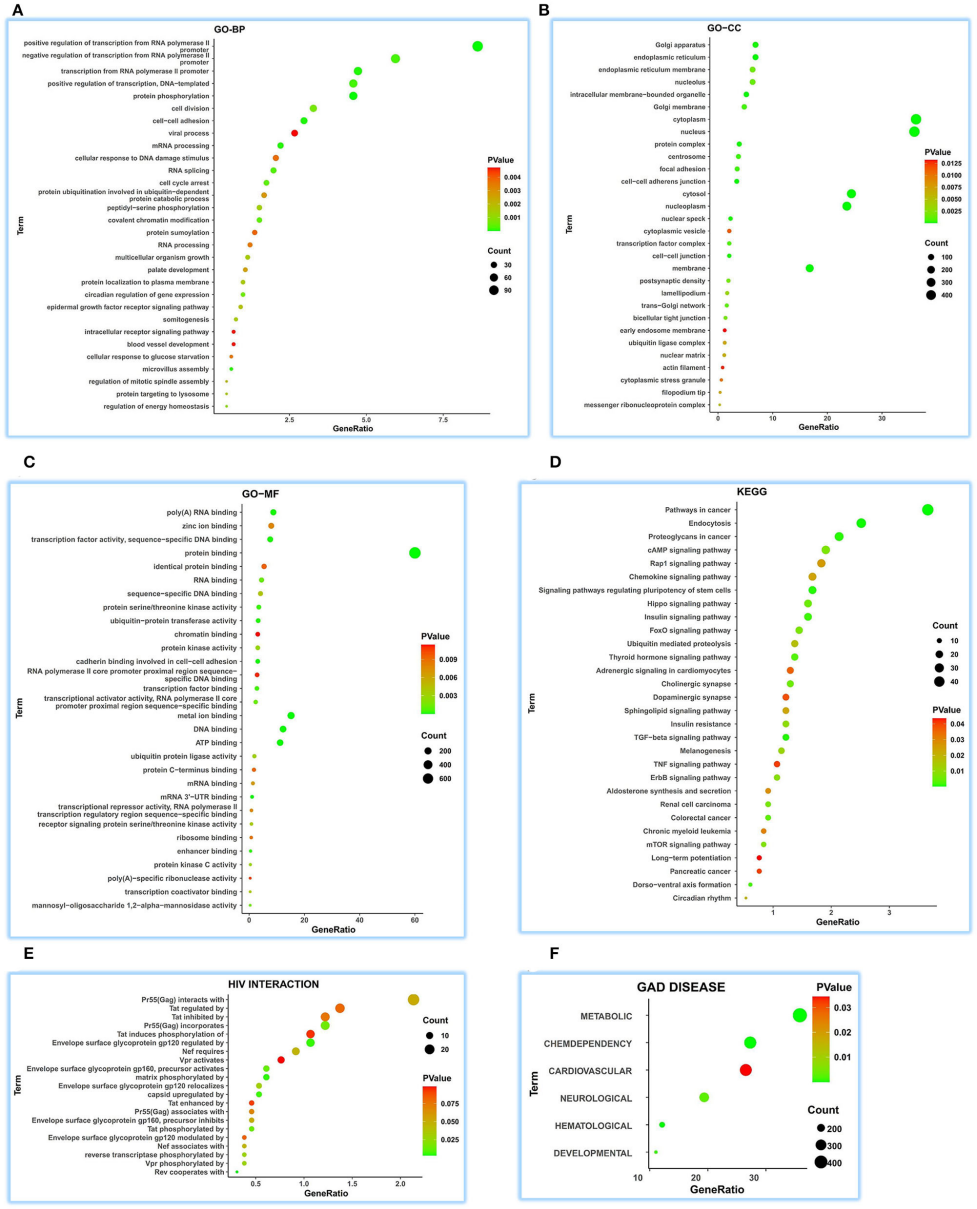
Comprehensive functional notes of ATP5MG by ceRNA analysis. Analyses of GO, KEGG, HIV interaction and GAD DISEASE were conducted using the DAVID tool based on 1326 ceRNA genes of ATP5MG in 32 human tissues. ATP5MG may take on various functions. **(A)** Functional terms for GO-BP. **(B)** Functional terms for GO-CC. **(C)** Functional terms for GO-MF. **(D)** Functional terms for KEGG pathway. **(E)** Functional terms for HIV interaction. **(F)** Functional terms for GAD DISEASE. GO, Gene Ontology; BP, biological process; CC, cellular component; MF, molecular function. KEGG, Kyoto Encyclopedia of Genes and Genomes.

We achieved 236 functional terms of GO-BP and found that ATP5MG may participate in the different biological processes, including “regulation of energy homeostasis,” “cellular response to glucose starvation,” “blood vessel development,” and “viral process” in the top 30 terms (Figure 3A). For GO-CC, 85 terms were found, which indicated that ATP5MG may be widely involved in different suborganelles (Figure 3B). GO-MF indicated 100 terms and found different molecular functions, such as “ATP binding” (Figure 3C). As for KEGG, we obtained 58 terms, including “insulin signaling pathway,” “cAMP signaling pathway,” “insulin resistance,” “chemokine signaling pathway,” “chronic myeloid leukemia,” “adrenergic signaling in cardiomyocytes,” and “TNF signaling pathway” in the top 30 terms (Figure 3D). HIV infection was further analyzed, and the involvement of ATP5MG in infection events with 21 terms was upheld (Figure 3E). Major functions of ATP5MG were further verified using GAD DISEASE analysis (Figure 3F), in which, we achieved 62 functional disease annotations and mainly grouped them into “CHEMDEPENDENCY,” “METABOLIC,” “HEMATOLOGIC,” “DEVELOPMENTAL,” “NEUROLOGICAL,” and “CARDIOVASCULAR.”

It can be seen from the above ceRNA analysis that ATP5MG-related functions are extensive, including metabolism, cardiovascular event, energy regulation, and inflammation/infection.

### Functional Notes of Intersection Genes Between CoRNA and CeRNA Genes of ATP5MG

**Figure 4:**
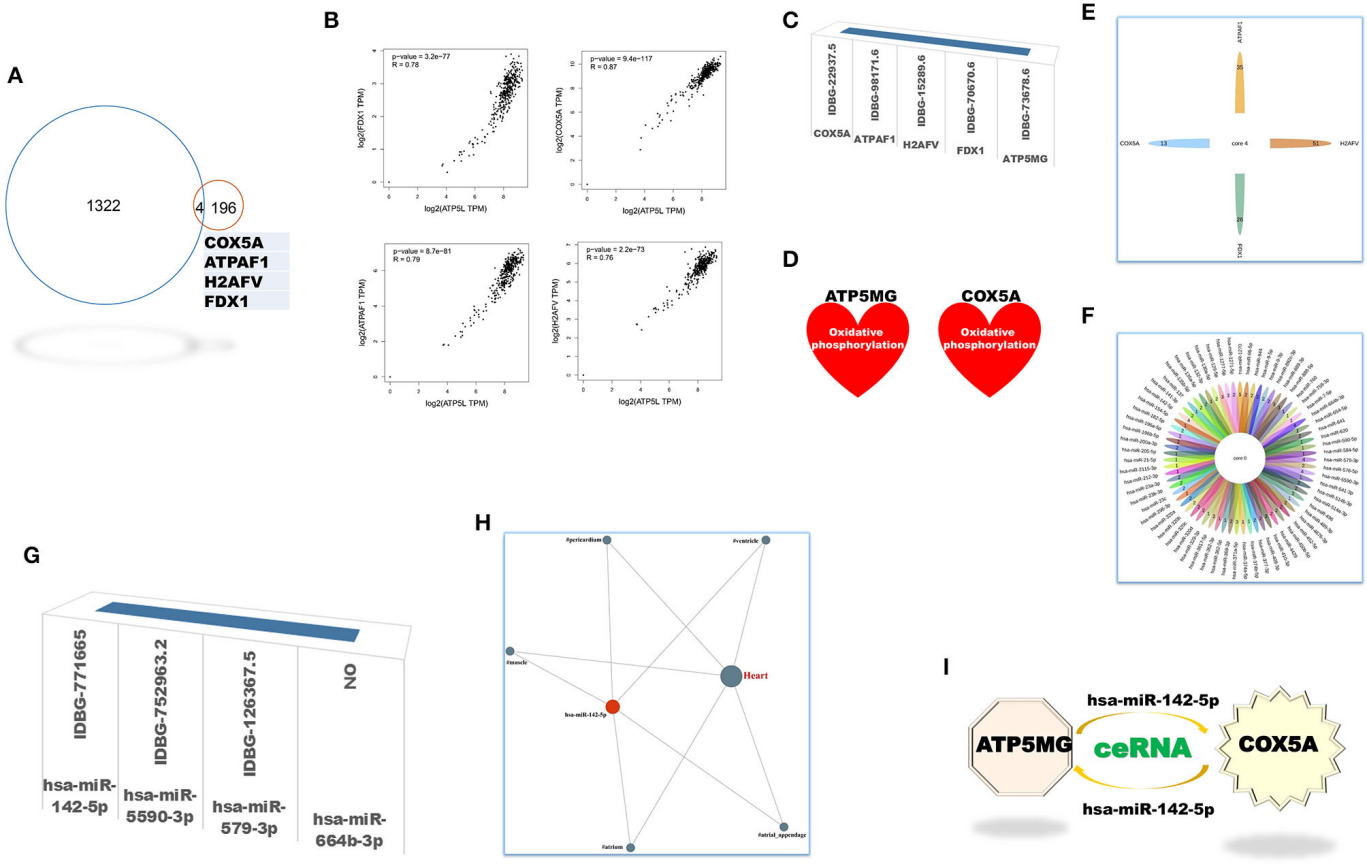
Intersection genes between coRNA and ceRNA genes of ATP5MG. Only four sharing coding genes were dug out for coexpression gene sets and ceRNA gene sets of ATP5MG. These four cross coding genes shared four miRNAs. Metabolic and immune annotations were recovered and verified metabolic and immune roles of one coding gene and one miRNA for ATP5MG. **(A)** Genes numbers of distinct datasets. **(B)** Expression relations between ATP5MG and the intersection genes in the heart. **(C)** Four intersection genes besides ATP5MG were annotated in innateDB. **(D)** COX5A, and ATP5MG were annotated in the metabolic database. **(E)** miRNA gene numbers of four intersection genes. **(F)** Intersected miRNAs of four intersection genes. **(G)** Three intersection miRNAs except hsa-miR-664b-3p were annotated in innateDB. **(H)** Regulation network of hsamiR-142-5p in the heart. **(I)** ATP5MG-centered ceRNA subnetwork.

The coRNA and ceRNA gene sets of ATP5MG were recruited to dig intersection genes, and only four cross coding genes were obtained (Figure 4A). PCC analysis identified high expression relationship indexes between ATP5MG and the intersection genes in the heart (Figure 4B). Tissue expression profiling of ATP5MG at nucleic acid and protein levels showed its extensive expression in distinct tissues, including high expression in the heart (Supplementary Figure 1). Tissue expression profiling of these four cross genes at protein levels indicated that only four genes except FDX1 exhibited extensive expression in distinct tissues, including the heart (Supplementary Figure 2). Cell type expression profiling of ATP5MG and these four genes in the heart revealed their shared four cell types, including cardiomyocytes and macrophages, which presented almost the same expression pattern (Supplementary Figure 3). Furthermore, we analyzed immune cell type specificity for the expression of the four intersected coding genes and ATP5MG, and their extensive expression was found in different immune cell types (Supplementary Figure 4). For all five genes, functional notes in KOBAS were analyzed for the KEGG pathway, and GO-related annotations were retrieved in innateDB (Supplementary Figure 5). It was revealed that ATP5MG was directly concerned with mitochondrial ATP production and metabolic pathway (Supplementary Figure 5A). COX5A functions in mitochondrial ATP production, metabolic pathway, and cardiac muscle contraction (Supplementary Figure 5B). Fewer feature notes of ATPAT1 (Supplementary Figure 5C) and H2AFV (Supplementary Figure 5D) were discovered. FDX1 had mitochondrial and metabolic roles (Supplementary Figure 5E). Recovering these five coding genes in innateDB identified their registration names (Figure 4C). But only ATP5MG and COX5A can be indexed in the metabolic database, indicating the involvement of “oxidative phosphorylation” (Figure 4D). From the above overall consideration, the ideal coding gene to form the ceRNA subnetwork of ATP5MG is COX5A.

Next, we set out to mine intersection miRNAs. COX5A can bind 13 miRNAs, ATPAF1 35 miRNAs, H2AFV 51 miRNAs, and FDX1 26 miRNAs (Figure 4E). All these four genes shared four miRNAs, namely, hsa-miR-142-5p, hsa-miR-5590-3p, hsa-miR-579-3p, and hsa-miR-664b-3p (Figure 4F). Except for hsa-miR-664b-3p, the other three miRNAs were recorded in innateDB as innate immune genes (Figure 4G). Furthermore, we profiled tissue expression of all four intersection miRNAs and found that, among them, hsa-miR-142-5p exhibited high and extensive expression in distinct parts of the heart, with the highest in the heart atrium (Supplementary Figure 6). Expression network analysis verified the expression of all parts of the heart for hsa-miR-142-5p (Figure 4H). From the above comprehensive consideration, the ideal miRNA to form the ceRNA subnetwork of ATP5MG is hsa-miR-142-5p.

Until now, by dissecting expressional and functional comments on intersection coding genes and miRNAs, we reached the target ceRNA subnetwork formed by ATP5MG, hsa-miR-142-5p, and COX5A (Figure 4I).

### Extensive Functions of ATP5MG by InterRNA Dissection

We fetched 507 interRNA human genes for functional comments using DAVID, which was built on RNA-RNA interaction of ENCORI.

**Figure 5:**
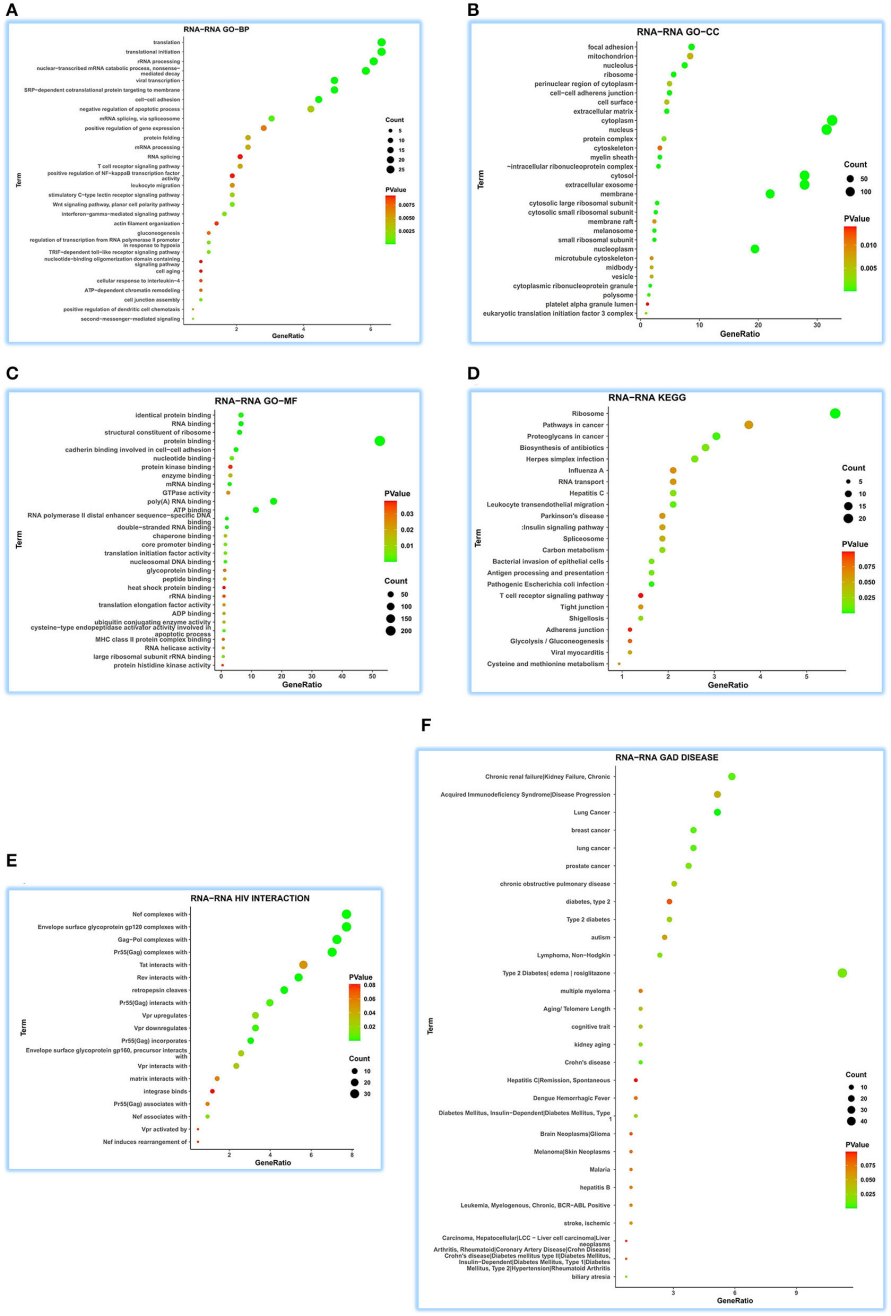
Extensive function annotations of ATP5MG by RNA-RNA interaction analysis. DAVID tool was enlisted to analyze GO, KEGG, HIV interaction, and GAD DISEASE based on 507 genes that can regulate ATP5MG by RNA-RNA interaction in 32 human tissues. The top 30 terms for each group were visualized. ATP5MG may function diversely. **(A)** Function annotations for GO-BP. **(B)** Function annotations for GO-CC. **(C)** Function annotations for GO-MF. **(D)** Function annotations for KEGG pathway. **(E)** Function annotations for HIV interaction. **(F)** Function annotations for GAD DISEASE. GO, Gene Ontology; BP, biological process; CC, cellular component; MF, molecular function. KEGG, Kyoto Encyclopedia of Genes and Genomes.

As for GO-BP, we obtained 199 function terms and found that ATP5MG may be related to different biological processes, including “viral transcription,” “interferon-gamma-mediated signaling pathway,” “T-cell receptor signaling pathway,” “leukocyte migration,” “gluconeogenesis,” “ATP-dependent chromatin remodeling,” “cellular response to interleukin-4,” and “positive regulation of NF-kappaB transcription factor activity” (Figure 5A). GO-CC saw 50 terms, showing its wide expression in different cell parts (Figure 5B). For GO-MF analysis, 43 terms were gained and different molecular functions were found, such as “ATP binding” and “MHC class II protein complex binding” (Figure 5C). When performing KEGG analysis, we acquired 23 terms, including metabolic, myocardic, and insulin signaling pathways, besides several infection-/inflammation-related annotations, such as “pathogenic *Escherichia coli* infection,” “leukocyte transendothelial migration,” “antigen processing and presentation,” “bacterial invasion of epithelial cells,” “herpes simplex infection,” “biosynthesis of antibiotics,” “hepatitis C,” “shigellosis,” “viral myocarditis,” “influenza A,” and “T-cell receptor signaling pathway” (Figure 5D).

Further analysis of HIV infection for 19 terms verified the broad involvement of ATP5MG in infection events (Figure 5E). ATP5MG-related extensive functions were further confirmed using GAD DISEASE analysis (Figure 5F), in which, we achieved 34 functional disease annotations, including “type 2 diabetes” and “lymphoma, non-Hodgkin,” “diabetes mellitus, type 1,” “chronic obstructive pulmonary disease,” “acquired immunodeficiency syndrome,” “stroke, ischemic,” “leukemia, myelogenous, chronic, BCR-ABL-Positive,” “hepatitis B,” and “hepatitis C.”

From the above interRNA dissection, it can be noted that ATP5MG regulates diabetes and myocarditis and has a focus on functions on immune/inflammation.

### Biological Roles of Intersection Genes Between InterRNA and CoRNA Genes of ATP5MG

**Figure 6:**
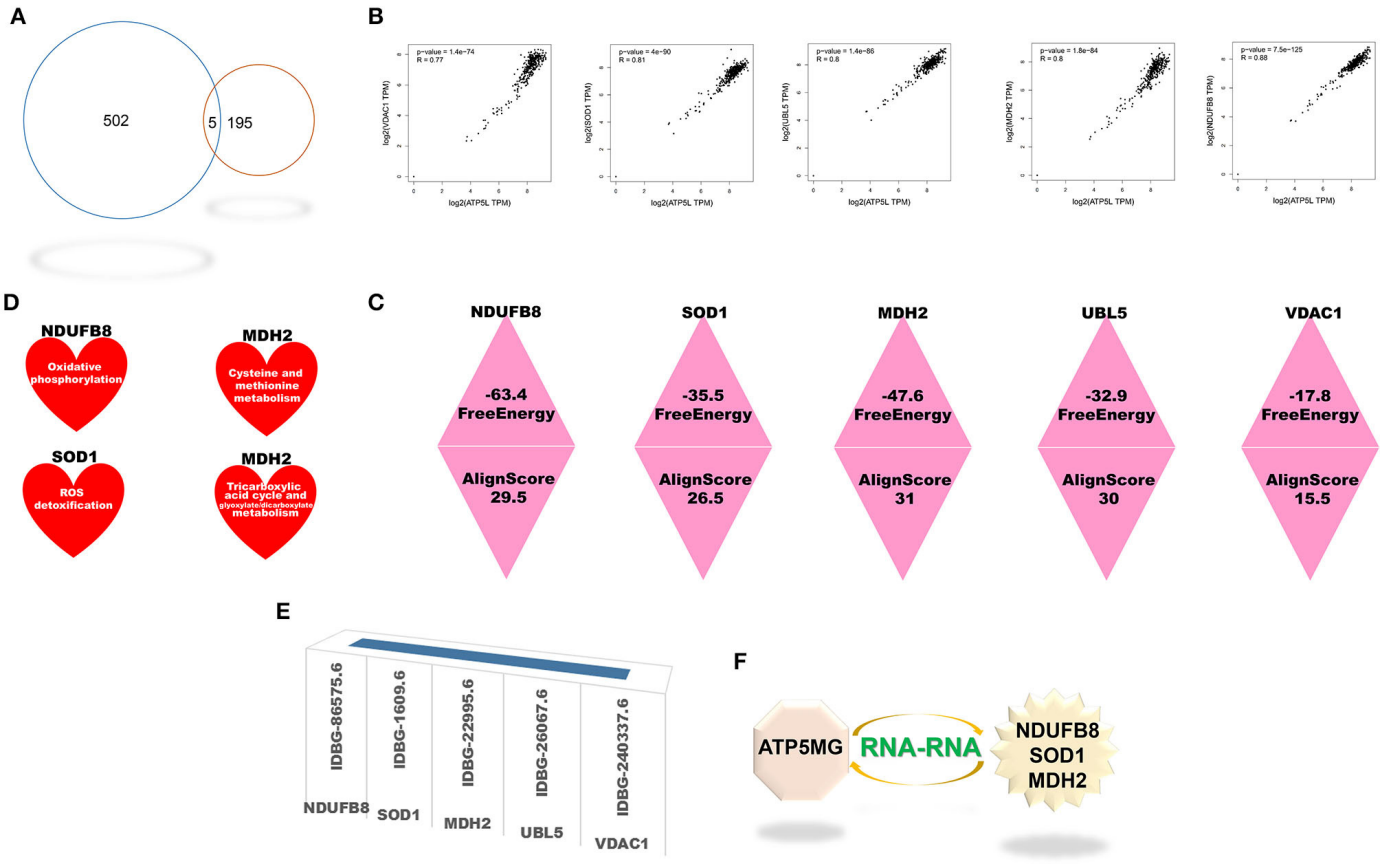
Intersection genes between coexpression and RNA-RNA-interacting genes of ATP5MG. Five intersection genes were mined for coexpression and RNA-RNA interaction gene sets of ATP5MG. The strength of the interaction is assessed. Metabolic and immune annotations were retrieved. **(A)** Genes numbers of distinct datasets. **(B)** Expression relations between ATP5MG and intersections. **(C)** Strength of the interplay of five intersection genes. **(D)** NDUFB8, SOD1, and MDH were annotated in the metabolic database. **(E)** Five cross genes were registered in innateDB. **(F)** ATP5MG-interplayed gene subnetwork.

Five cross genes, namely, NDUFB8, SOD1, MDH2, UBL5, and VDAC1, were gained based on dissecting interRNA and coRNA genes of ATP5MG (Figure 6A). ATP5MG and the five genes had high expression relation indexes in the heart (Figure 6B). The five genes had very low free energy and very high alignment scores, except for VDAC1 (Figure 6C). Tissue expression, cell expression, and immune and metabolic queries were dissected to select the ideal genes to form a subnetwork of RNA–RNA interaction.

Tissue expression profiling of these four genes at protein levels disclosed extensive expression in distinct tissues, including the heart (Supplementary Figure 7). However, UBL5 data are unavailable. Cell type expression profiling of these five genes in the heart uncovered their shared four cell types, including cardiomyocytes and macrophages (Supplementary Figure 8). Profiling distinct immune cell types for the five intersected coding genes found their extensive expression in immune cells (Supplementary Figure 9). Querying the metabolic database uncovered the involvement of NDUFB8, SOD1, and MDH2 in the metabolism (Figure 6D).

All five genes were identified in the InnateDB database (Figure 6E), where GO-related functional notes were extracted. It was shown that NDUFB8 was associated with mitochondrial ATP production and metabolic pathway (Supplementary Figure 10A). SOD1, a famous antioxidant, functions diversely, including mitochondrial ATP production, metabolic regulation, heart and vascular role, and immune/inflammation regulation (Supplementary Figures 10B–D). MDH2 showed mitochondrial and metabolic roles (Supplementary Figure 10E). Fewer function notes of UBL5 (Supplementary Figure 10F) and VDAC1 (Supplementary Figure 10G) were recovered.

As the results shown above, taken together, the ideal genes to form the interRNA subnetwork of ATP5MG are NDUFB8, SOD1, and MDH2 (Figure 6F).

### Environmental Exposures and Clinical Drugs Targeting Intersection Genes of ATP5MG-Circled Network

**Figure 7:**
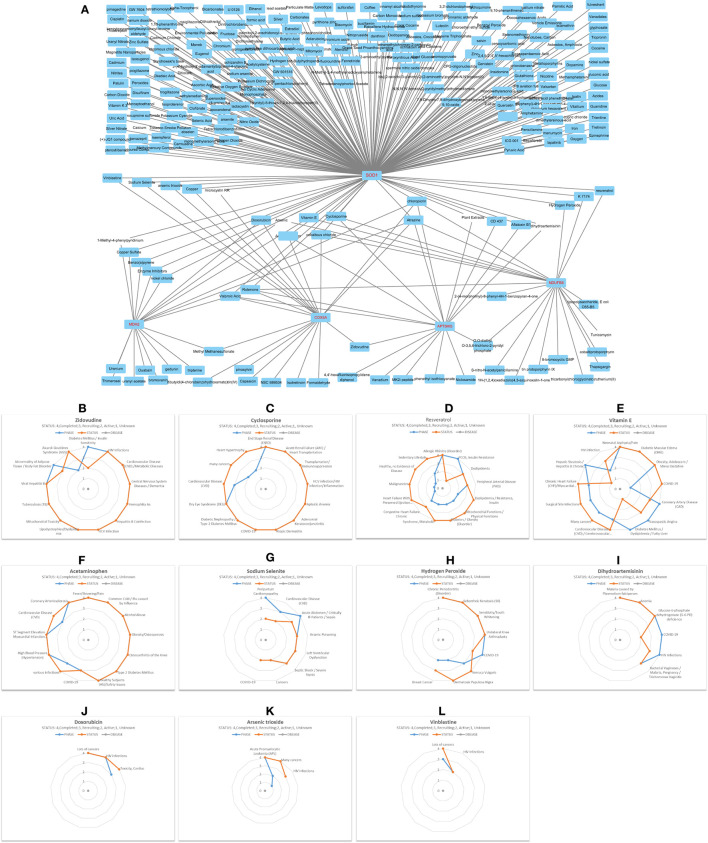
Decipher chemicals (drugs) targeted critical network genes. **(A)** Mine 251 chemicals targeted the five coding genes in the ATP5MG-related network. **(B)** Clinical trials of Zidovudine. **(C)** Clinical trials of Cyclosporine. **(D)** Clinical trials of Resveratrol. **(E)** Clinical trials of Vitamin E. **(F)** Clinical trials of Acetaminophen. **(G)** Clinical trials of Sodium selenite. **(H)** Clinical trials of Hydrogen peroxide. **(I)** Clinical trials of Dihydroartemisinin. **(J)** Clinical trials of Doxorubicin. **(K)** Clinical trials of Arsenic trioxide. **(L)** Clinical trials of Vinblastine.

Through all sorts of screening, we harvested a six-gene network comprising ceRNA and interRNA subnetworks. The ceRNA subnetwork comprises ATP5MG, hsa-miR-142-5p, and COX5A, while the interRNA subnetwork includes other three genes, namely, NDUFB8, SOD1, and MDH2. This network is viewed as a mediator of MetS-CVD. To ascertain their practical significance, we first employed environmental exposures from the CTD database to query chemicals perturbing our network. As miRNA information is unavailable in this database, we just checked the five coding genes, namely, ATP5MG, COX5A, NDUFB8, SOD1, and MDH2. A total of 251 chemicals were identified to target them (Figure 7A). Among them, five chemicals affect the expression of MDH2 and SOD1, one affects MDH2 and COX5A, one affects COX5A and ATP5MG, one affects ATP5MG and NDUFB8, four affect ATP5MG and SOD1, three affect NDUFB8 and SOD1, and five affect COX5A and SOD1. There were six chemicals targeted to three genes, two targeted to four genes, and two targeted to five genes.

Then, we conducted a match analysis using DrugBank, a distinguished clinical drug database, to verify whether some of these chemicals can be prescribed as drugs to treat clinical diseases. Advanced clinical trials of some of these drugs are visualized here. Zidovudine exhibited phase 4 for diabetes mellitus/insulin sensitivity, CVD/metabolic diseases, and various inflammation/infection-related diseases (Figure 7B). Cyclosporine exhibited phase 4 for diabetic nephropathy/type 2 diabetes mellitus, HCV infection/HIV infection/inflammation, and COVID-19, and phase 3 for cardiovascular disease (CVD), phase 2 for heart hypertrophy (Figure 7C). Resveratrol owned phase 4 for “allergic rhinitis (disorder)” and “PCOS, insulin resistance,” phase 3 for “dyslipidemia” and “peripheral arterial disease (PAD),” phase 2 for “mitochondrial functions/physical functions,” “diabetes/obesity (disorder), “syndrome, metabolic,” and “congestive heart failure chronic,” and phase 1 for “heart failure with preserved ejection fraction (HFpEF)/heart failure, diastolic/high blood pressure (hypertension)/hypertensive heart disease/stress oxidative” (Figure 7D). Vitamin E possessed phase 4 for “diabetic macular edema (DME),” “obesity, adolescent/stress oxidative,” “coronary artery disease (CAD),” “vasospastic angina,” “diabetes mellitus/dyslipidemia/fatty liver,” and “hepatic steatosis/hepatitis B chronic infection,” phase 3 for “cardiovascular disease (CVD)/cerebrovascular diseases,” numerous cancers, “surgical site infections,” “chronic heart failure (CHF)/myocardial infarction (ischemia),” phase 2 for HIV infection, and phase 1 for COVID-19 (Figure 7E). Acetaminophen had many clinical trials at phase 4 for “obesity/osteoporosis,” “osteoarthritis of the knee,” “type 2 diabetes mellitus,” “high blood pressure (hypertension),” “ST segment elevation” myocardial infarction (STEMI),” “coronary arteriosclerosis,” phase 3 for COVID-19, different infections, and CVD (Figure 7F).

Sodium selenite had phase 4 for peripartum cardiomyopathy and sepsis, phase 3 for CVD, arsenic poisoning, and left ventricular dysfunction, and phase 2 for COVID-19 (Figure 7G). Hydrogen peroxide saw phase 4 for inflammation or infections, including COVID-19 (Figure 7H). Dihydroartemisinin is a famous drug with phase 4 clinical trials for glucose-6-phosphate dehydrogenase (G-6-PD) deficiency and different infections, including COVID-19 (Figure 7I). Doxorubicin can treat many cancers and HIV infections, and it had phase 3 for “Toxicity, Cardiac” (Figure 7J). Arsenic trioxide (Figure 7K) and vinblastine (Figure 7L) were often used to deal with different cancers, and they both had clinical trials for HIV infections.

It can be seen from the above that five drugs, namely, zidovudine, cyclosporine, resveratrol, vitamin E, and acetaminophen, have an obvious ability to treat inflammation-/immunity-related diseases, metabolism-related diseases, and CVD, strongly supporting our idea. The fringe benefits include six drugs, namely, cyclosporine, vitamin E, acetaminophen, sodium selenite, hydrogen peroxide, and dihydroartemisinin, which can be administered to treat COVID-19.

**Figure 8:**
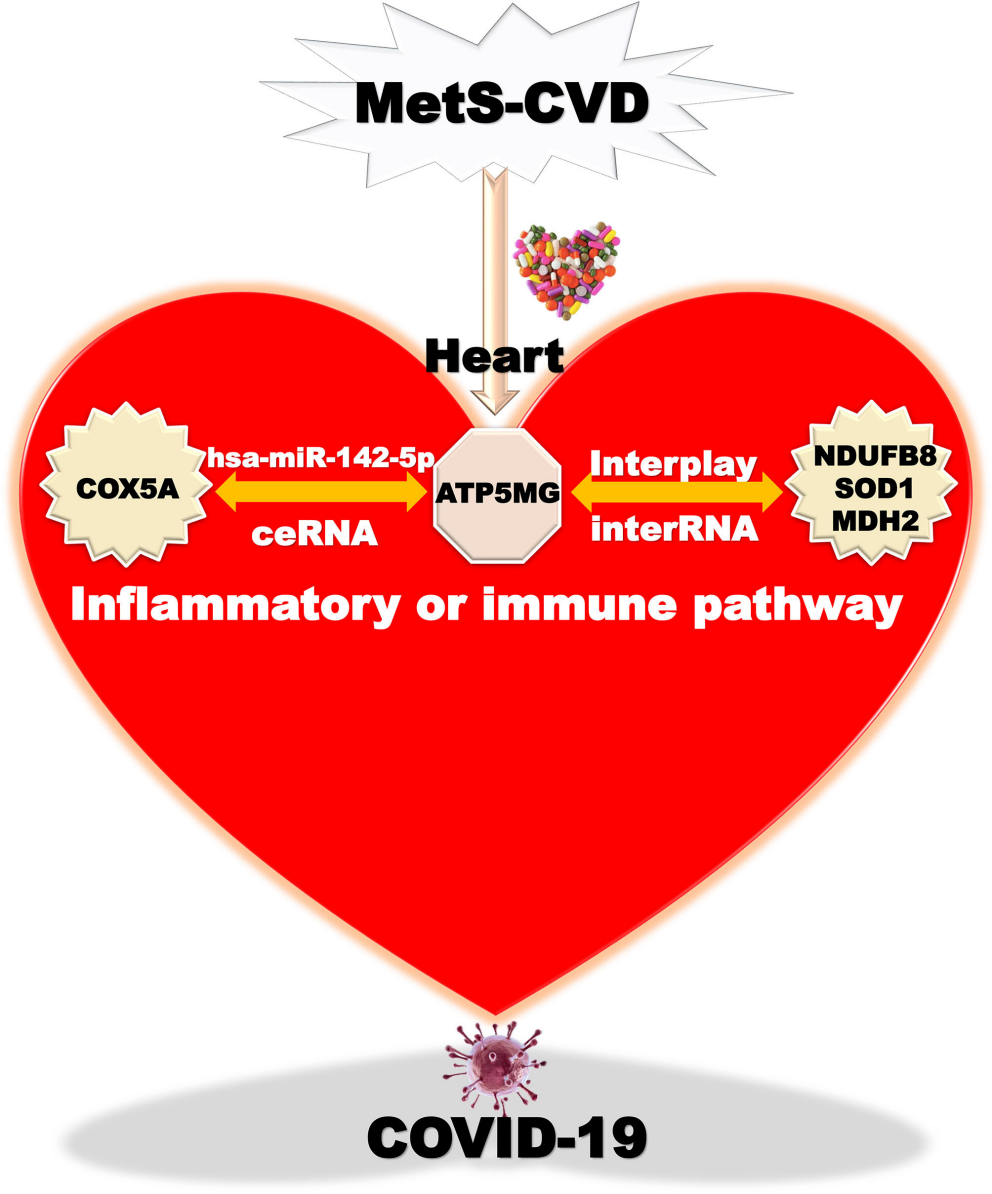
Taking home message on ATP5MG-centered network. Inflammatory or immune pathway mediated by ATP5MG-centered network in MetS-CVD. Chemicals or drugs target this network formed by the ceRNA subnetwork and interRNA subnetwork. This network may be involved in COVID-19.

Overall, we finally harvest an underlying mechanism mediated by the ATP5MG-centered inflammatory/immune pathway (Figure 8). Chemicals or drugs affect Mets-CVD by targeting this network comprising ceRNA and interRNA subnetworks, which include one miRNA and four coding genes.

## Discussion

In this study, we screened for the ATP5MG-oriented molecular network for MetS-CVD. Our molecular network relied on the strength of ceRNA and interRNA. ceRNA is not an RNA but a new mechanism of gene expression regulation suggested recently (31). ceRNA shows the mutual regulation of two long RNA molecules by competing for binding to shared microRNAs, a family of small non-coding RNAs that are essential regulators of gene expression (32). Essentially, ceRNA is still an RNA-RNA interaction, while interRNA means a direct interaction of long RNAs. Reading this new regulatory network will produce novel insights into MetS-CVD. Notably, there is no distinction between “MetS and CVD” and MetS-CVD because these two diseases are closely coupled and pathologically unclear diseases. Still, there is no distinction between MetS and obesity/diabetes/other metabolism-related diseases because they are often inextricably linked. They were viewed as metabolic diseases to analyze identically.

Recent reports on ceRNA presented its involvement in inflammation/immunity in CVDs. Time-ordered ceRNA networks are constructed in ischemic and dilated cardiomyopathy (33). The ceRNA network controls inflammation through tanshinone IIA in atherosclerosis (34), affects metabolic and proinflammatory responses in coronary artery disease (CAD) (35), and regulates inflammation and cell proliferation in remote ischemic preconditioning of myocardial ischemia-reperfusion injury (36). NEAT1-formed ceRNA participates in endothelial dysfunction by regulating inflammation in vein graft failure (37). GATA5-mediated ceRNA adjusts cardiac conduction block through an inflammatory process (38). H19-related ceRNA regulates sepsis myocardial dysfunction (39) and ameliorates myocardial injury and maladaptive cardiac remodeling partially by adjusting inflammatory response (40). ceRNA constructed by CDKN2B-AS1 inhibits VSMC proliferation by inhibiting the inflammatory factors (41). ceRNA generated by PEAMIR restrains the inflammatory response in myocardial ischemia/reperfusion injury exacerbated by PM2.5 exposure (42). XIST, as a ceRNA participator, impacts inflammation and pyroptosis in atrial fibrillation (43). From all these reviewed, it can be seen that, our ceRNA subnetwork, ATP5MG/hsa-miR142-5p/COX5A, focused on metabolic and inflammatory signals, is a novel mechanism, which may provide new insights into CVD (Figure 4I).

We can also find some studies on ceRNA when we retrieve metabolic diseases. miR-146a-5p-mediated ceRNAs regulate inflammation in diabetic peripheral neuropathy (44). Paternal folate modulates lipid and glucose metabolism in broiler offspring through the ceRNA mechanism (45). Metabolism-related ceRNA may act as a predictor of the survival outcomes of patients with osteosarcoma (46). CASC2/miR-9-5p/PPARγ alleviates the high glucose-induced cell injury in diabetes nephropathy (47). In polycystic ovary syndrome (PCOS), a ceRNA network is constructed (48), most ceRNA axes are closely related to steroid biosynthesis and metabolic pathways (49), and PWRN2-mediated ceRNA may regulate oocyte nuclear maturation (50). Exosomal circLDLR/miR-1294/CYP19A1 represses estradiol manufacture in PCOS (51). Key ceRNA pairs in metabolic pathways may participate in right ventricular dysfunction (52). circRNA/lncRNA/miRNA/mRNA inhibits macrophage inflammation in type 1 diabetes mellitus (T1DM) (53). AC063977.6/miR-338-3p/PFKFB2 may regulate metabolic event during Intervertebral disc degeneration (IDD) pathogenesis (54). LncRNAs-based ceRNA reflects in circulating extracellular vesicles in human MetS (55). Based on the above, from the MetS perspective, our ceRNA network is still a new network.

Relatively, the involvement of ceRNA in energy metabolism in metabolic diseases has been less reported. A study showed possible energy metabolic regulation by ceRNA under grass-fed and grain-fed regimens in angus beef cattle (56). Another research indicates that ANRIL improves the mitochondrial function of hepatocellular carcinoma by regulating the miR-199a-5p/ARL2 axis (57). The study of energy metabolism's involvement in the ceRNA mechanism of metabolic diseases is just beginning, and our investigation is timely.

The role of RNA-RNA interaction in CVD has been little examined. lncRNA TBX5-AS1:2 is involved in tetralogy of Fallot, the most common complex congenital heart disease, by affecting the mRNA stability of TBX5 through RNA-RNA interaction (58). Likewise, there are few examinations of the involvement of RNA–RNA interaction in metabolic diseases. LINC01537 stabilizes PDE2A mRNA to promote its expression through RNA–RNA interaction regulating energy metabolism in lung cancer (59). Therefore, it can be seen that our interRNA subnetwork, ATP5MG-NDUFB8/SOD1/MDH2, regulated by metabolic and inflammatory signals, is a novel mechanism that may produce new insights into MetS-CVD (Figure 6F).

For a novel molecular network, experimental explorations using cellular and animal models are required to verify the suggested mechanism. As we are unable to perform experimental operations, CTD and DrugBank were employed to mine drugs to confirm the clinical values of our network. As is known, only if a gene is biologically critical, it can be investigated in clinical trials. If the clinical drug information cannot be found, the network will be of no value or needs future information. Crucially, such drugs have to be able to treat inflammation/immunity-related diseases, also MetS and CVD. By wonderful good fortune, we mined much valuable drug information. Eleven valuable drugs were selected from these chemicals (Figures 7B–L). Among them, five drugs (i.e., zidovudine, cyclosporine, resveratrol, vitamin E, and acetaminophen) (Figures 7B–F) had the potential to treat inflammation/immunity-related diseases, also metabolism-related diseases and CVD, which give strong support to our purpose.

As presented in facts, combating COVID-19 is like fighting a series of wars, so COVID-19 is called WARS [58]. In this study, we mined six drugs (i.e., cyclosporine, vitamin E, acetaminophen, sodium selenite, hydrogen peroxide, and dihydroartemisinin), which can be conscribed to win the WARS (Figure 7), showing the value of our strategy in targeting inflammation or immunity-related pathway, and unexpected benefits of potential host-targeting antiviral strategies. Intriguingly, acetaminophen, hydrogen peroxide, and artemisinin (dihydroartemisinin) are all bitter medicines, which are the ligands of bitter taste receptors (60, 61). Host-directed therapy using bitter medicine (HDT-BM) was suggested to fight infection-related diseases, including WARS (62, 63), in which inflammatory or immune pathways are naturally involved. Just as the bitter medicine caffeine (62), acetaminophen, hydrogen peroxide, and artemisinin (dihydroartemisinin) may function broadly by targeting several molecules (mechanism of action in DrugBank), including bitter taste receptors, which showed the complicated regulatory mechanisms of gene expression. That means examining a network, not a single molecule, is of particular significance.

## Conclusion

In this bioinformatics examination using public big data, it was revealed that ATP5MG might form ceRNA with COX5A through hsa-miR-142-5p and interplay with NDUFB8, SOD1, and MDH2 by the RNA–RNA interaction under inflammation-/immunity-related scenes. We decoded clinical trials of five medicines from 251 chemicals targeting this network, including zidovudine, cyclosporine, resveratrol, vitamin E, and acetaminophen, which may be recruited to treat CVD, metabolic diseases, and inflammation-/immunity-related diseases; reasonably, they may also treat MetS-CVD. Ecstatically, we got a windfall, that is, cyclosporine, vitamin E, acetaminophen, sodium selenite, hydrogen peroxide, and dihydroartemisinin may be employed to deal with COVID-19. Thus, our study may provide basic and clinical insights into epigenetic network regulation for metabolism-immunity highly integrated diseases.

## Data Availability Statement

The datasets presented in this study can be found in online repositories. The names of the repository/repositories and accession number(s) can be found in the article/Supplementary Material.

## Author Contributions

XL: conception, design, and manuscript writing. LL, XZ, and JC: provision of study materials. LL: collection and assembly of data. All authors: administrative support, data analysis and interpretation, and final approval of manuscript.

## Funding

This study was supported by the Outstanding Leaders Training Program of Pudong Health Bureau of Shanghai (PWRl2018-02), the Pudong New Area Science and Technology Commission of Shanghai (PKJ2019-Y21 and PKJ2020-Y38), and the Shanghai Municipal Health Commission (202140467).

## Conflict of Interest

The authors declare that the research was conducted in the absence of any commercial or financial relationships that could be construed as a potential conflict of interest.

## Publisher's Note

All claims expressed in this article are solely those of the authors and do not necessarily represent those of their affiliated organizations, or those of the publisher, the editors and the reviewers. Any product that may be evaluated in this article, or claim that may be made by its manufacturer, is not guaranteed or endorsed by the publisher.
