# Supplementary material for: Potential of ATP5MG to Treat Metabolic Syndrome-Associated Cardiovascular Diseases
Source: Front Cardiovasc Med. 2022 Jul 22;9:921778. doi: 10.3389/fcvm.2022.921778 (PMC9355403; doi:10.3389/fcvm.2022.921778)
Supplement: Supplementary file 1 [file Data_Sheet_1.PDF]

SUPPL FIG.1

A

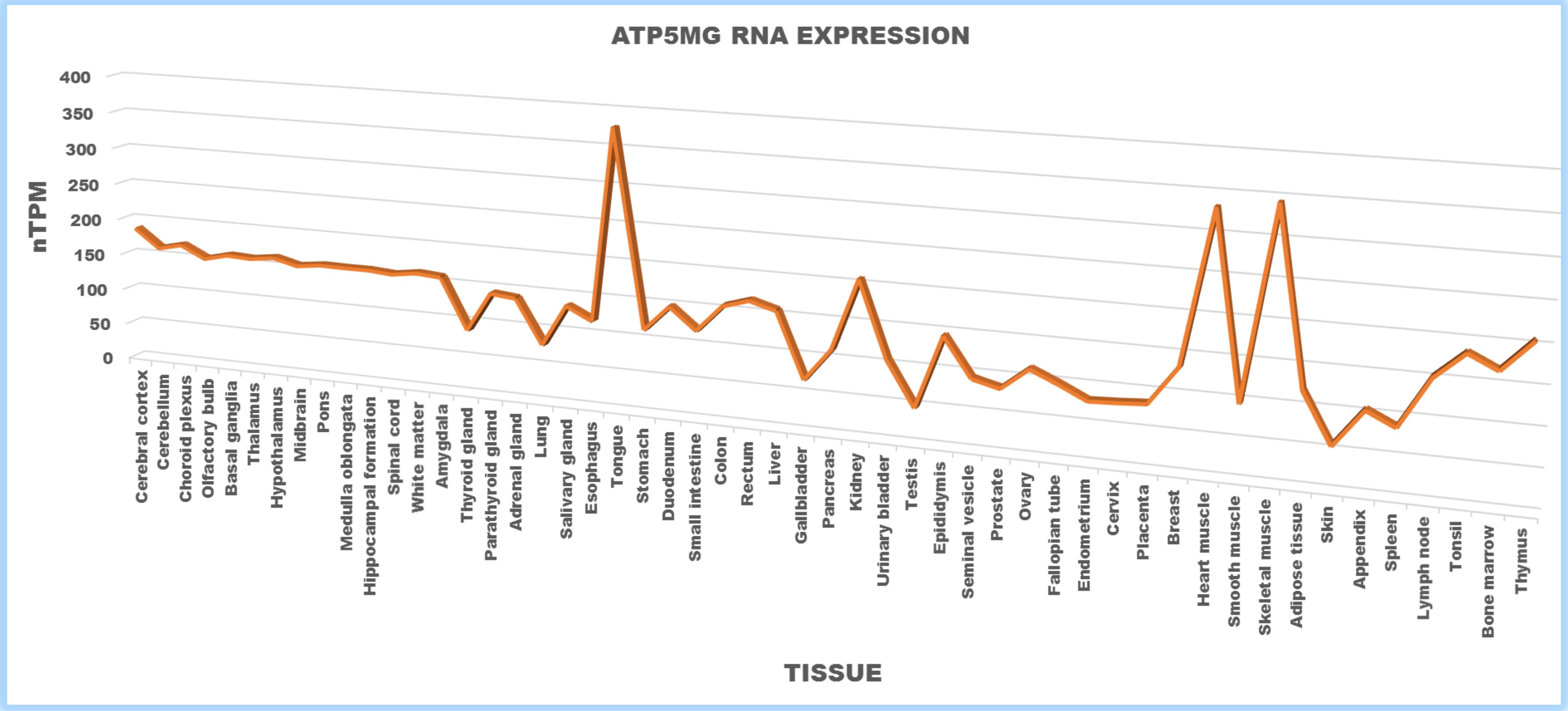

B

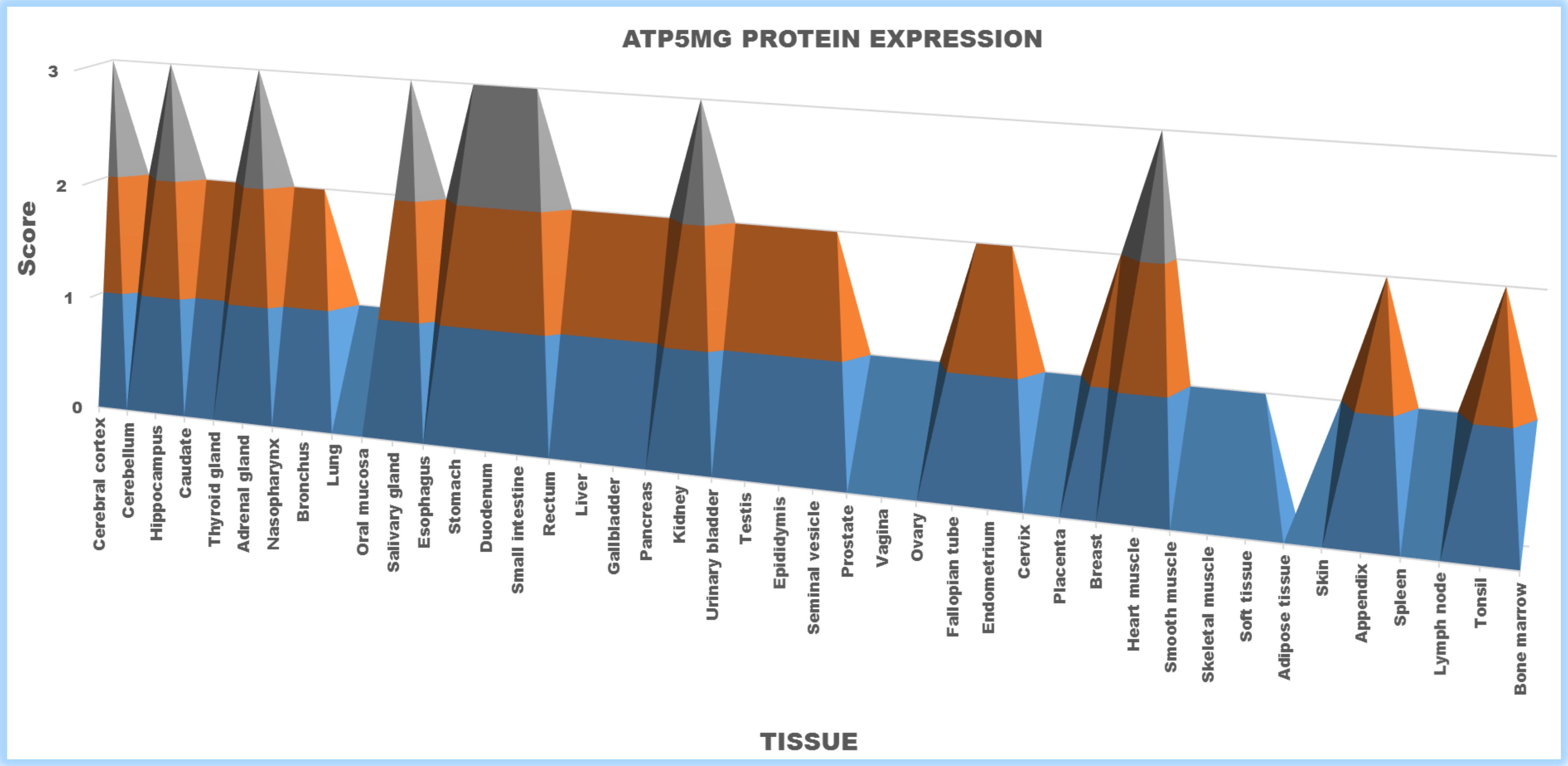

SUPPLE FIG. 1

Tissue expression profiling of ATP5MG

A, Tissue expressions of ATP5MG at the nucleic acid level. B, Tissue expressions of ATP5MG at the protein level.

SUPPL FIG.2

A

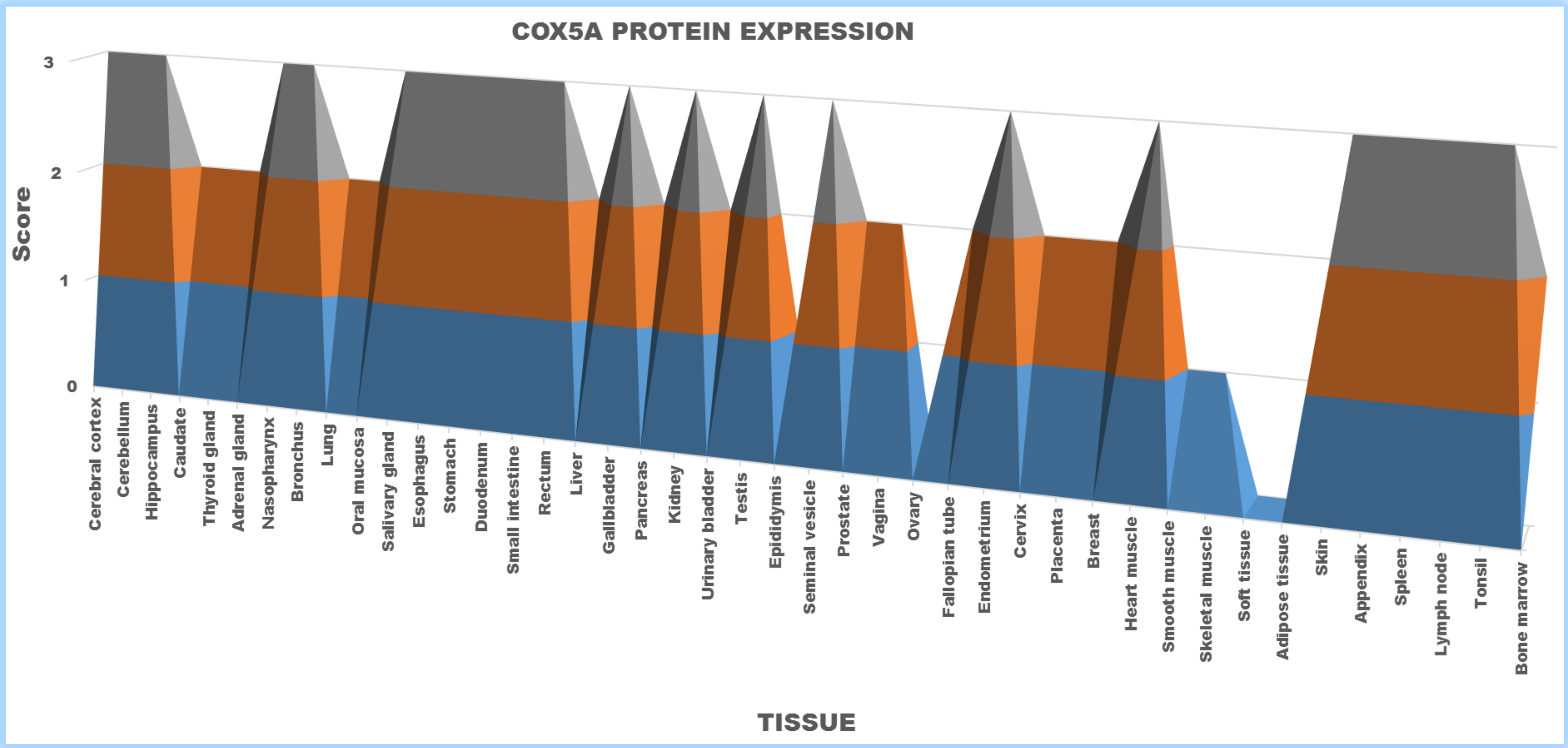

B

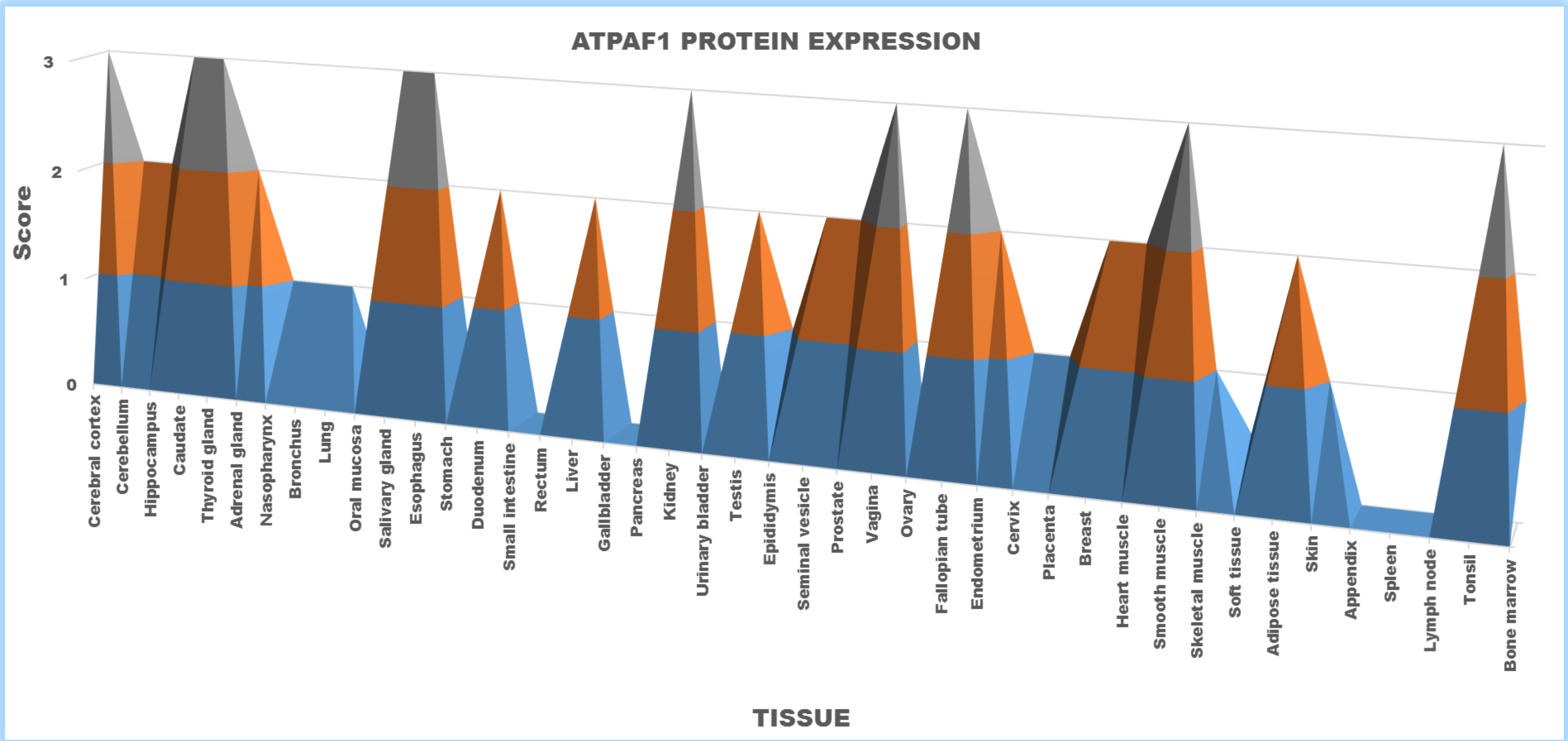

C

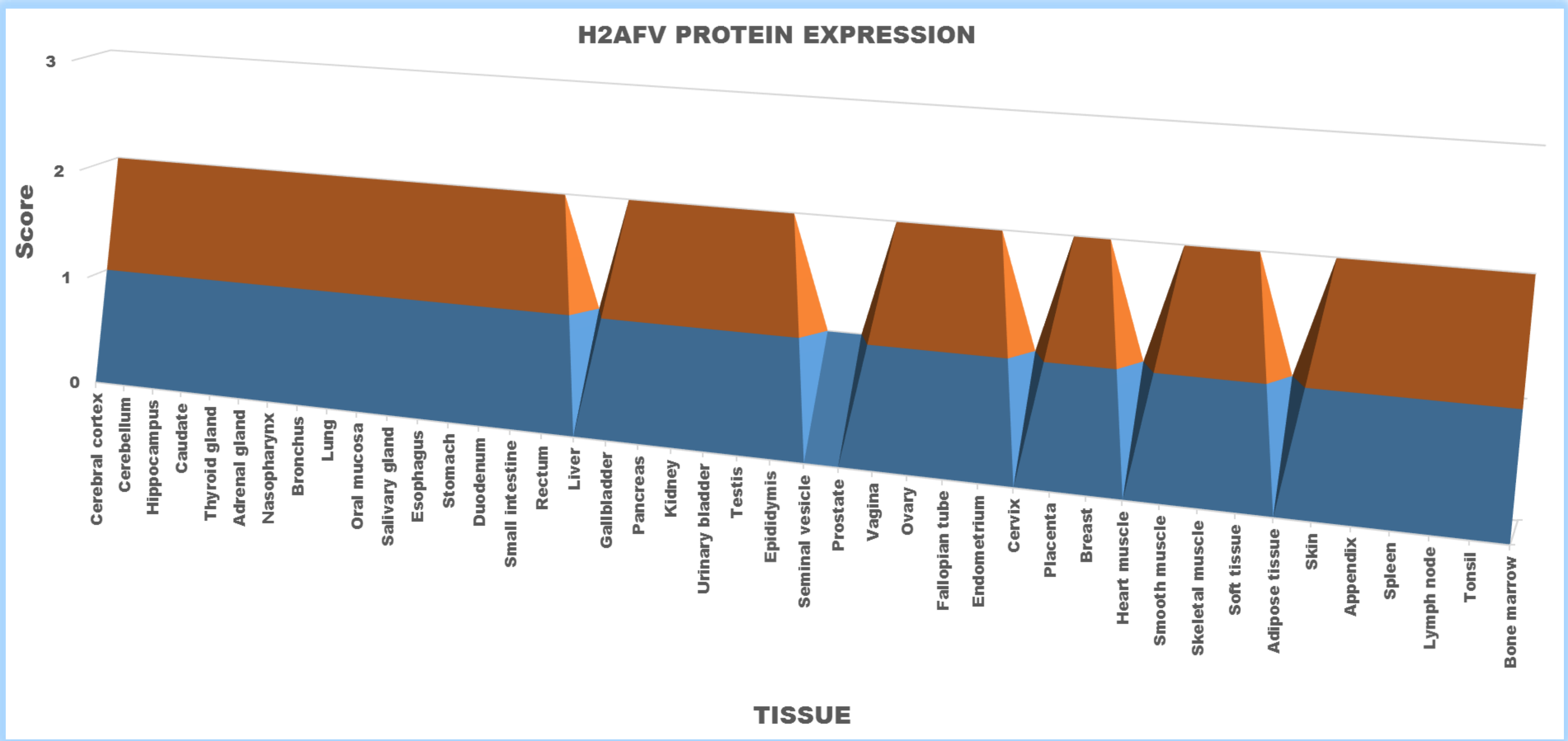

D

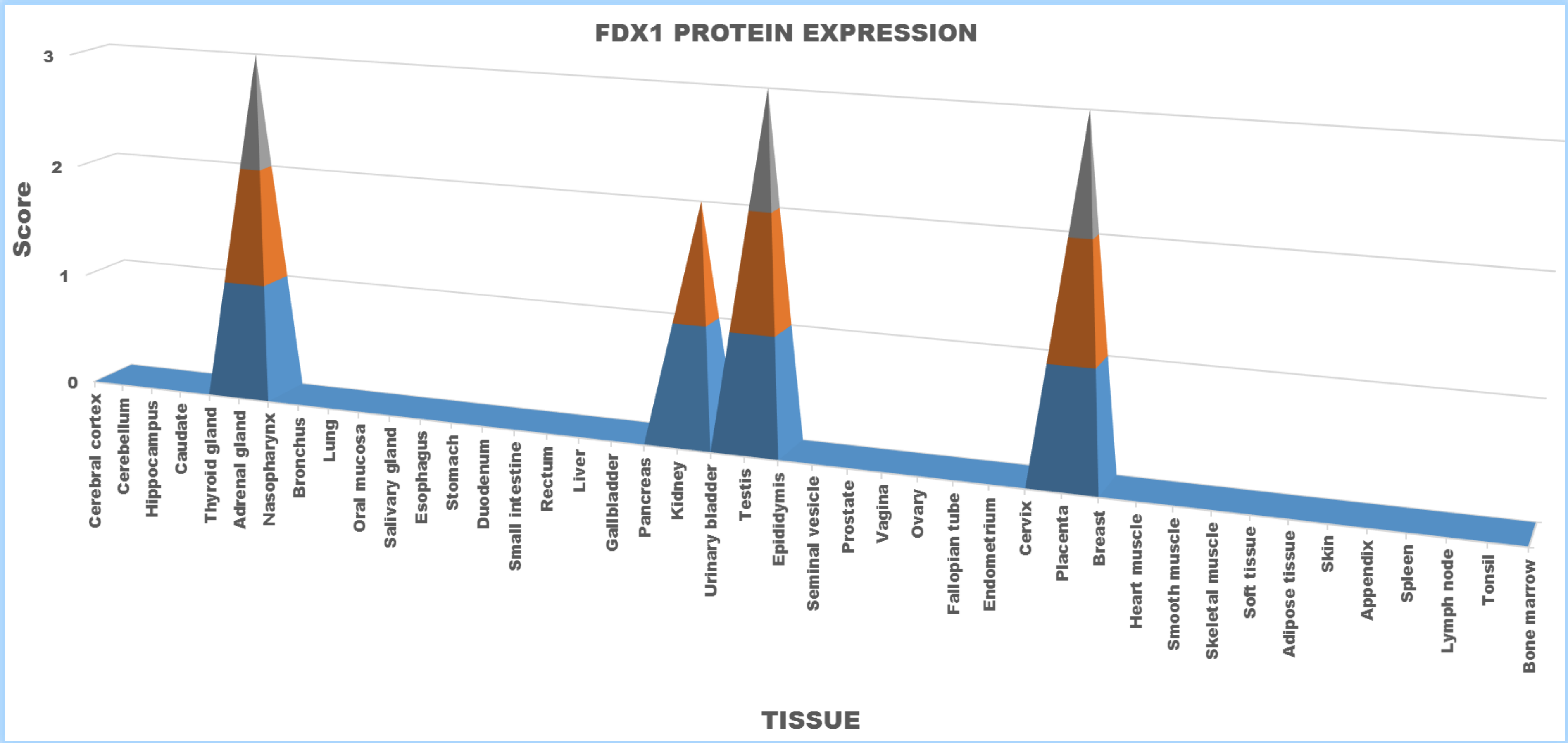

SUPPLE FIG. 2

Tissue expression profiling of the intersected coding genes of ATP5MG

A, Tissue expressions of COX5A. B, Tissue expressions of ATPAF1. C, Tissue expressions of H2AFV. D, Tissue expressions of FDX1.

SUPPL FIG.3

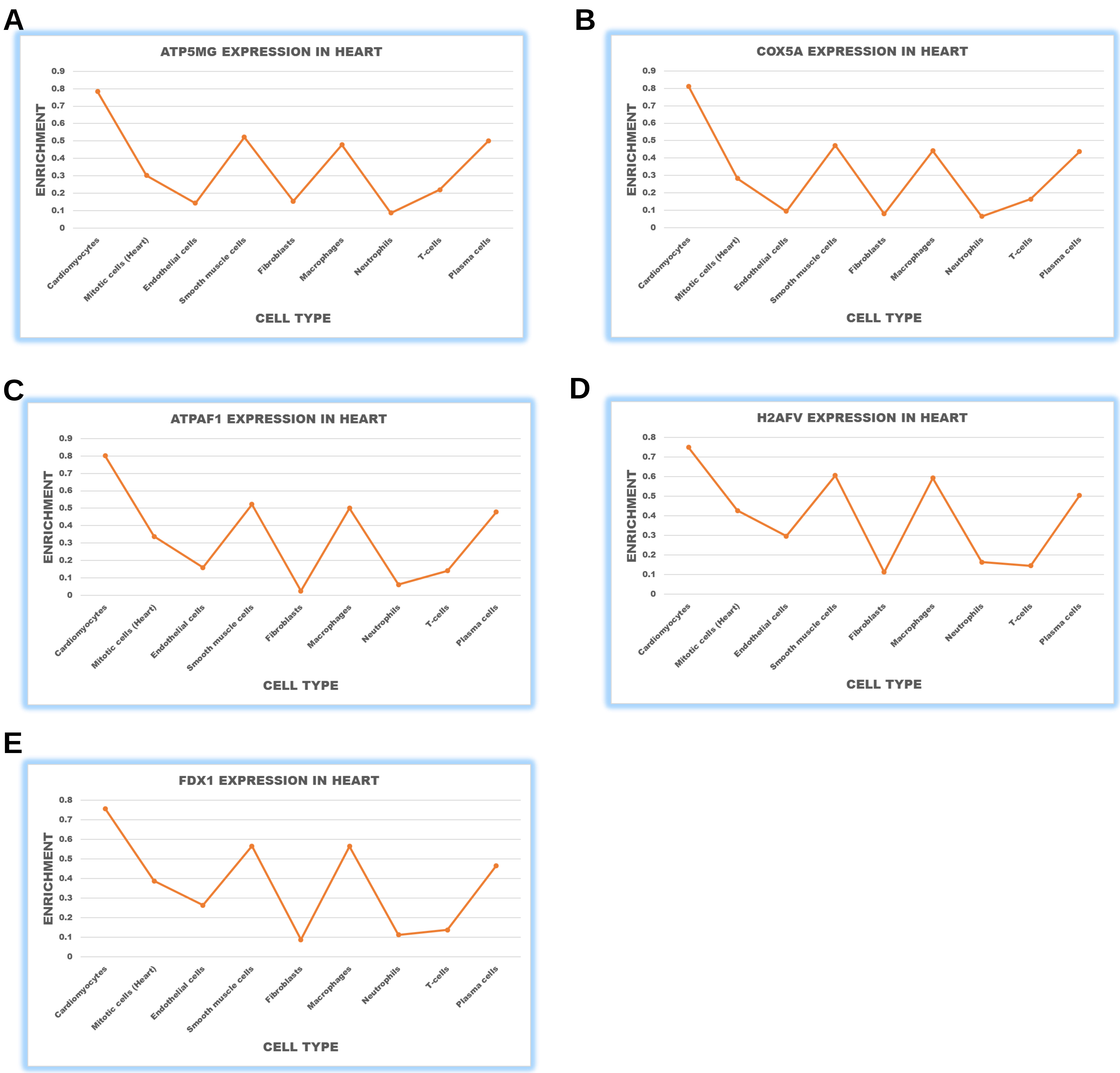
A
ATP5MG EXPRESSION IN HEART
B
COX5A EXPRESSION IN HEART
C
ATPAF1 EXPRESSION IN HEART
D
H2AFV EXPRESSION IN HEART
E
FDX1 EXPRESSION IN HEART
ENRICHMENT
CELL TYPE
0
0.1
0.2
0.3
0.4
0.5
0.6
0.7
0.8
0.9
Cardiomyocytes
Mitotic cells (Heart)
Endothelial cells
Smooth muscle cells
Fibroblasts
Macrophages
Neutrophils
T-cells
Plasma cells

SUPPLE FIG. 3

Cell expression profiling of ATP5MG and the intersected coding genes in the heart.

A, Cell expression of ATP5MG. B, Cell expression of COX5A. C, Cell expression of ATPAF1. D, Cell expression of H2AFV. E, Cell expression of FDX1.

SUPPL FIG.4

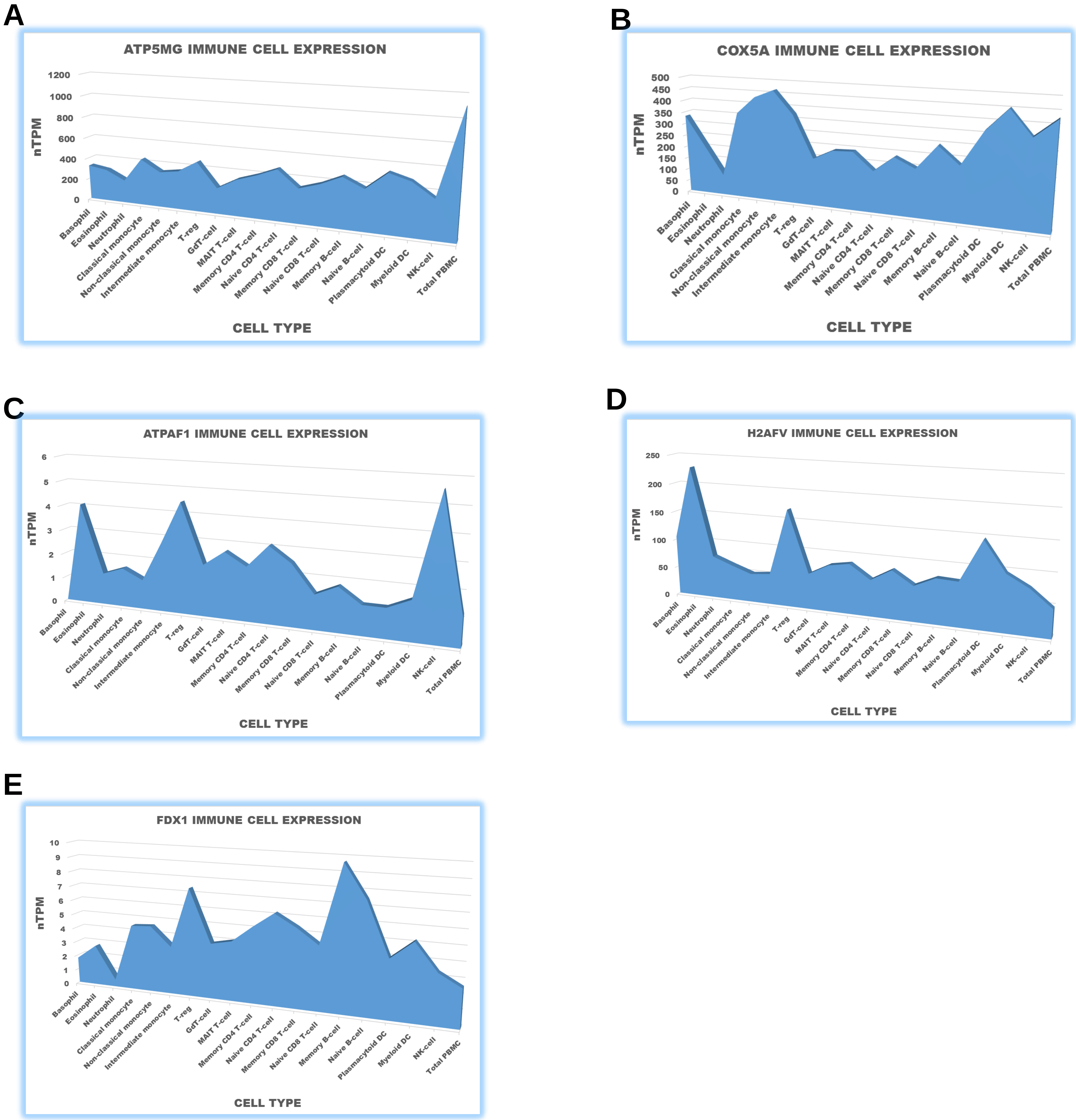
A
ATP5MG IMMUNE CELL EXPRESSION
nTPM
0
200
400
600
800
1000
1200
Basophil
Eosinophil
Neutrophil
Classical monocyte
Non-classical monocyte
Intermediate monocyte
T-reg
GdT-cell
MAIT T-cell
Memory CD4 T-cell
Naive CD4 T-cell
Memory CD8 T-cell
Naive CD8 T-cell
Memory B-cell
Naive B-cell
Plasmacytoid DC
Myeloid DC
NK-cell
Total PBMC
CELL TYPE
B
COX5A IMMUNE CELL EXPRESSION
nTPM
0
50
100
150
200
250
300
350
400
450
500
CELL TYPE
C
ATPAF1 IMMUNE CELL EXPRESSION
nTPM
0
1
2
3
4
5
6
CELL TYPE
D
H2AFV IMMUNE CELL EXPRESSION
nTPM
0
50
100
150
200
250
CELL TYPE
E
FDX1 IMMUNE CELL EXPRESSION
nTPM
0
1
2
3
4
5
6
7
8
9
10
CELL TYPE

SUPPLE FIG. 4

Analyzing immune cell-type specificity of the expression of ATP5MG and the four intersected coding genes

Extensive expressions in various immune cell types were found for the four intersected coding genes and ATP5MG. A, Expressions of ATP5MG. B, Expressions of COX5A. C, Expressions of ATPAF1. D, Expressions of H2AFV. E, Expressions of FDX1.

SUPPL FIG.5

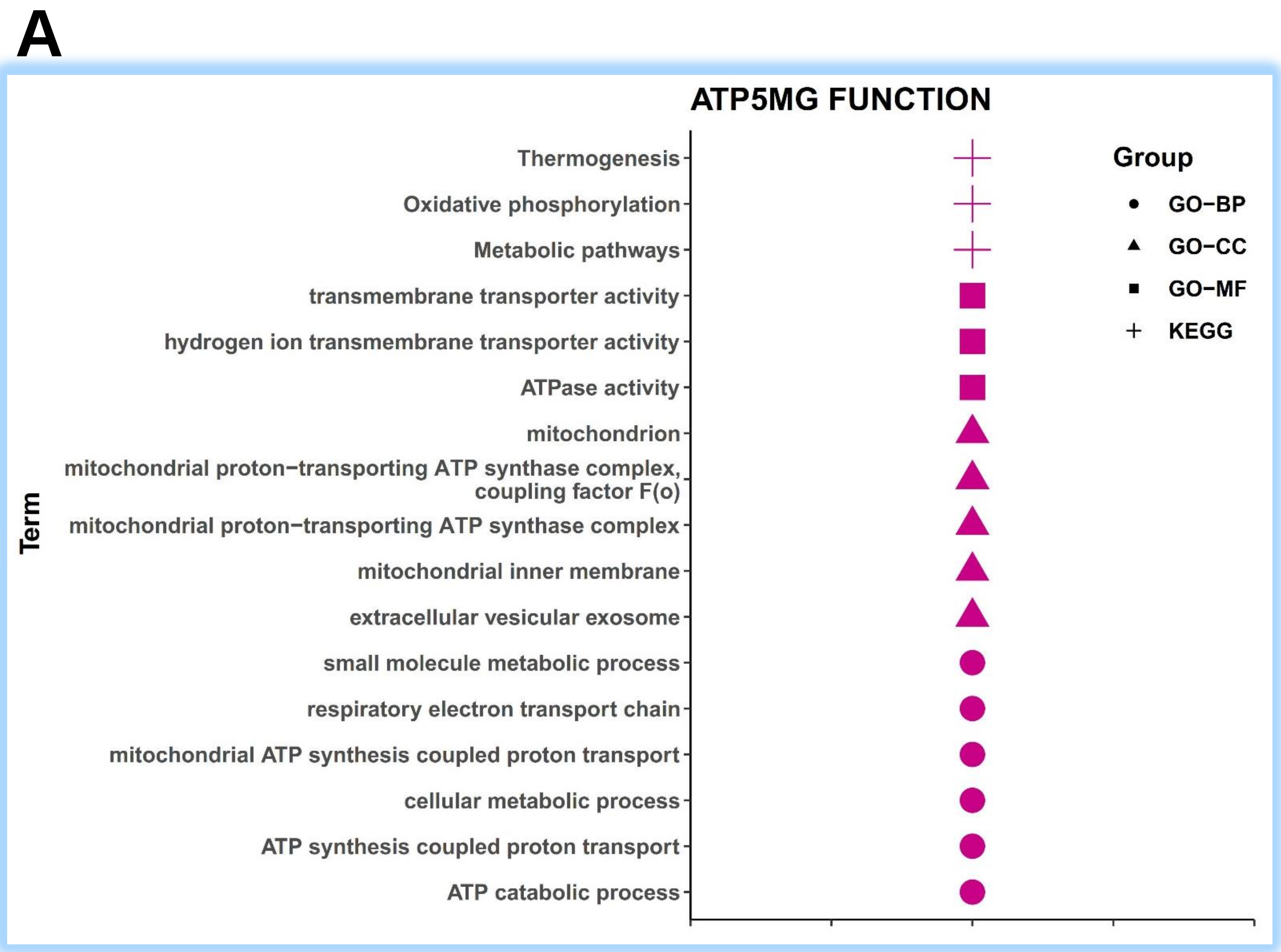
A
ATP5MG FUNCTION
Term
Thermogenesis
Oxidative phosphorylation
Metabolic pathways
transmembrane transporter activity
hydrogen ion transmembrane transporter activity
ATPase activity
mitochondrion
mitochondrial proton−transporting ATP synthase complex, coupling factor F(o)
mitochondrial proton−transporting ATP synthase complex
mitochondrial inner membrane
extracellular vesicular exosome
small molecule metabolic process
respiratory electron transport chain
mitochondrial ATP synthesis coupled proton transport
cellular metabolic process
ATP synthesis coupled proton transport
ATP catabolic process
Group
GO−BP
GO−CC
GO−MF
KEGG

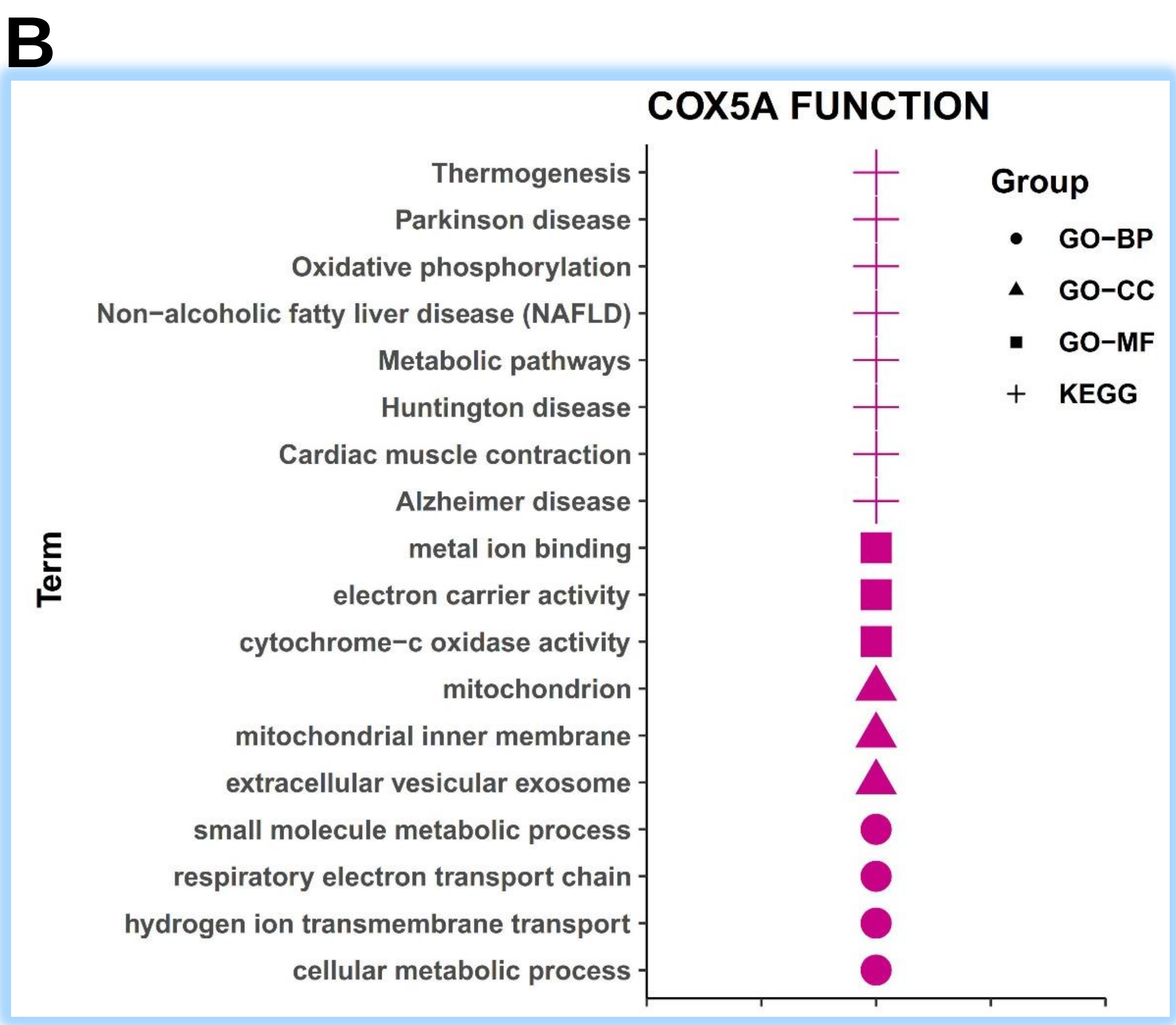
B
COX5A FUNCTION
Term
Thermogenesis
Parkinson disease
Oxidative phosphorylation
Non−alcoholic fatty liver disease (NAFLD)
Metabolic pathways
Huntington disease
Cardiac muscle contraction
Alzheimer disease
metal ion binding
electron carrier activity
cytochrome−c oxidase activity
mitochondrion
mitochondrial inner membrane
extracellular vesicular exosome
small molecule metabolic process
respiratory electron transport chain
hydrogen ion transmembrane transport
cellular metabolic process
Group
GO−BP
GO−CC
GO−MF
KEGG

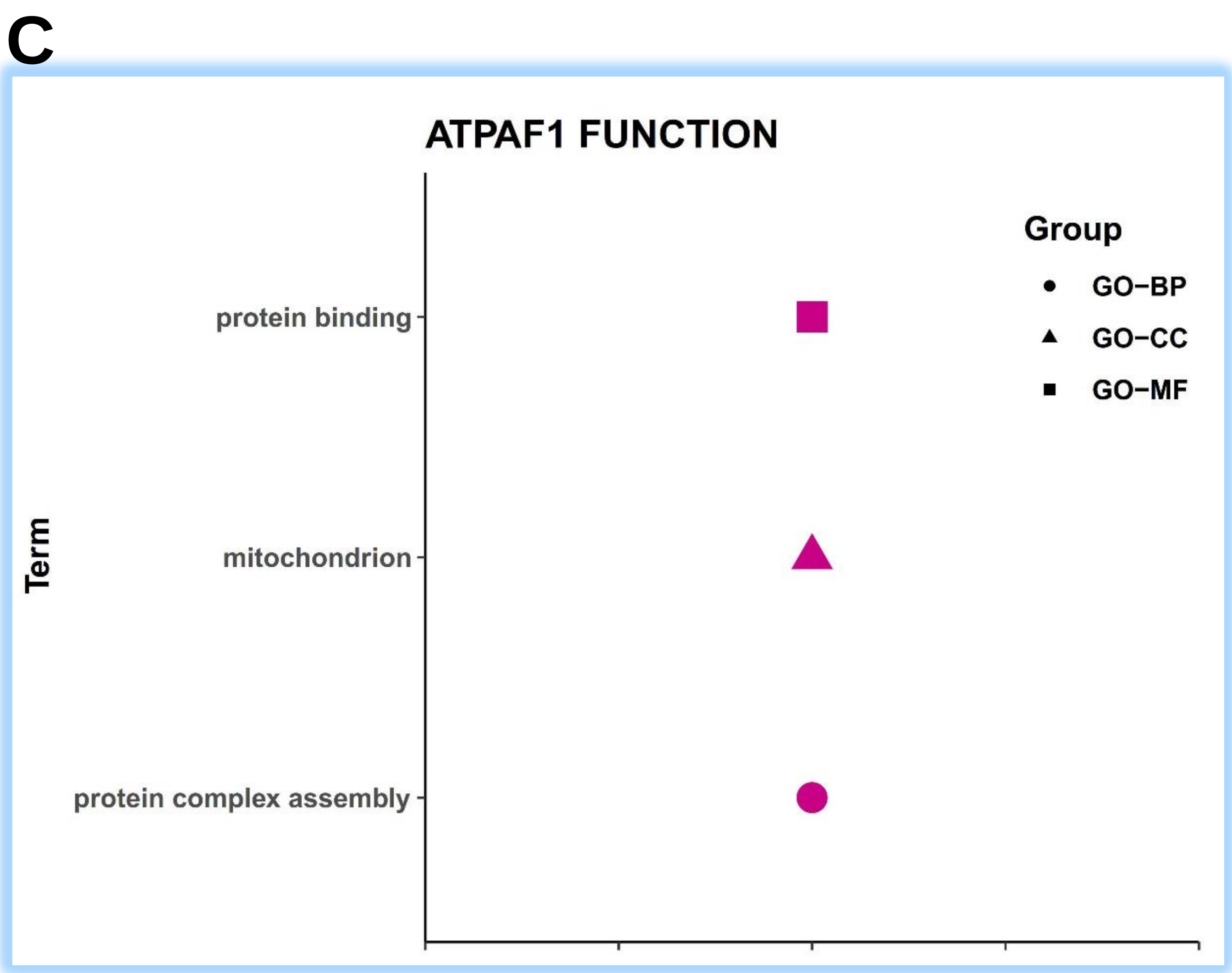
C
ATPAF1 FUNCTION
Term
protein binding
mitochondrion
protein complex assembly
Group
GO−BP
GO−CC
GO−MF

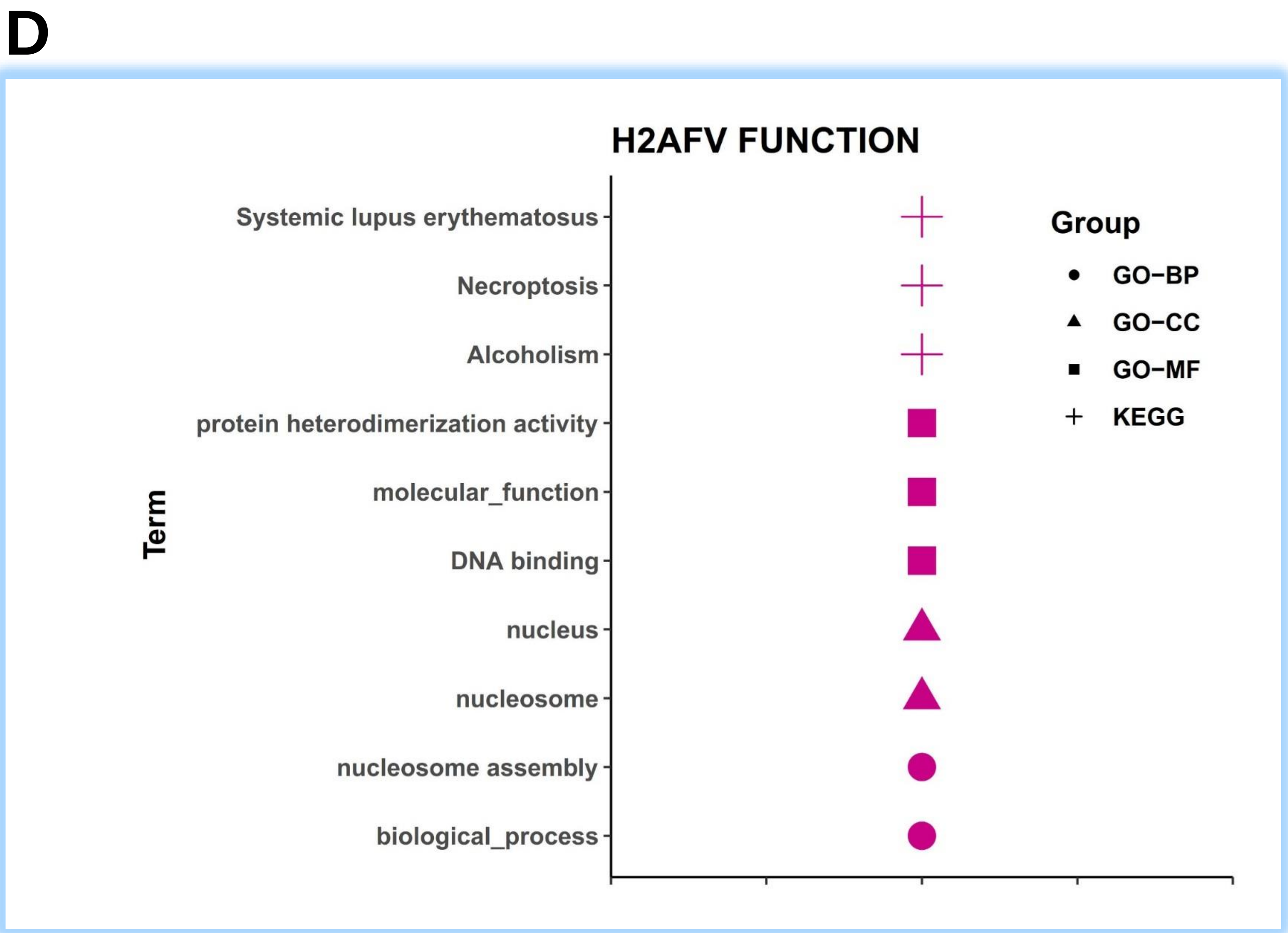
D
H2AFV FUNCTION
Term
Systemic lupus erythematosus
Necroptosis
Alcoholism
protein heterodimerization activity
molecular_function
DNA binding
nucleus
nucleosome
nucleosome assembly
biological_process
Group
GO−BP
GO−CC
GO−MF
KEGG

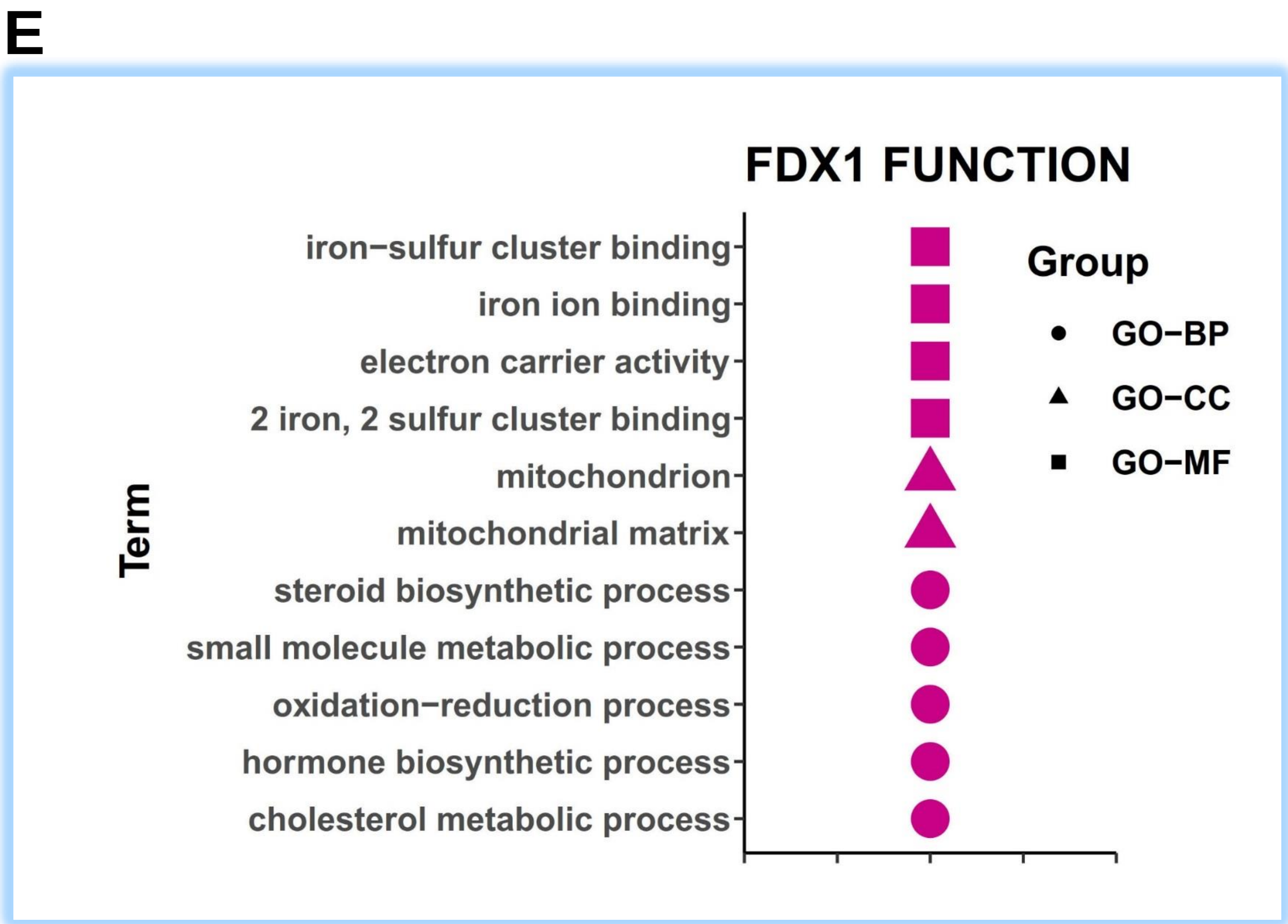
E
FDX1 FUNCTION
Term
iron−sulfur cluster binding
iron ion binding
electron carrier activity
2 iron, 2 sulfur cluster binding
mitochondrion
mitochondrial matrix
steroid biosynthetic process
small molecule metabolic process
oxidation−reduction process
hormone biosynthetic process
cholesterol metabolic process
Group
GO−BP
GO−CC
GO−MF

SUPPLE FIG. 5 Functional comments of ATP5MG and four intersected coding genes

Annotations of GO and KEGG were recovered and visualized. A, Functional terms for ATP5MG. B, Functional terms for COX5A. C, Functional terms for ATPAF1. D, Functional terms for H2AFV. E, Functional terms for FDX1. GO, Gene Ontology; BP, biological process; CC, cellular component; MF, molecular function. KEGG, Kyoto Encyclopedia of Genes and Genomes.

SUPPL FIG.6

A

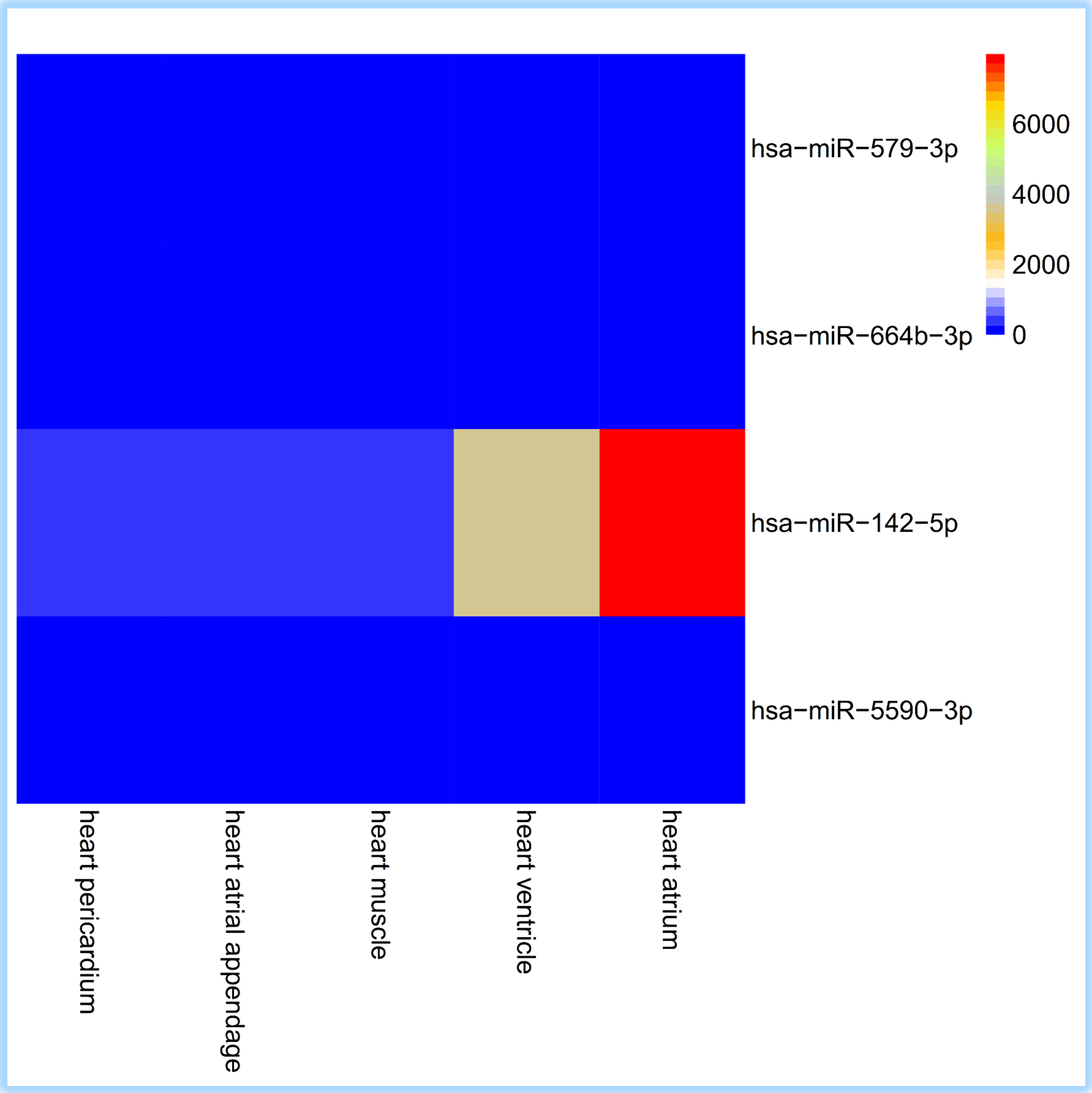

B

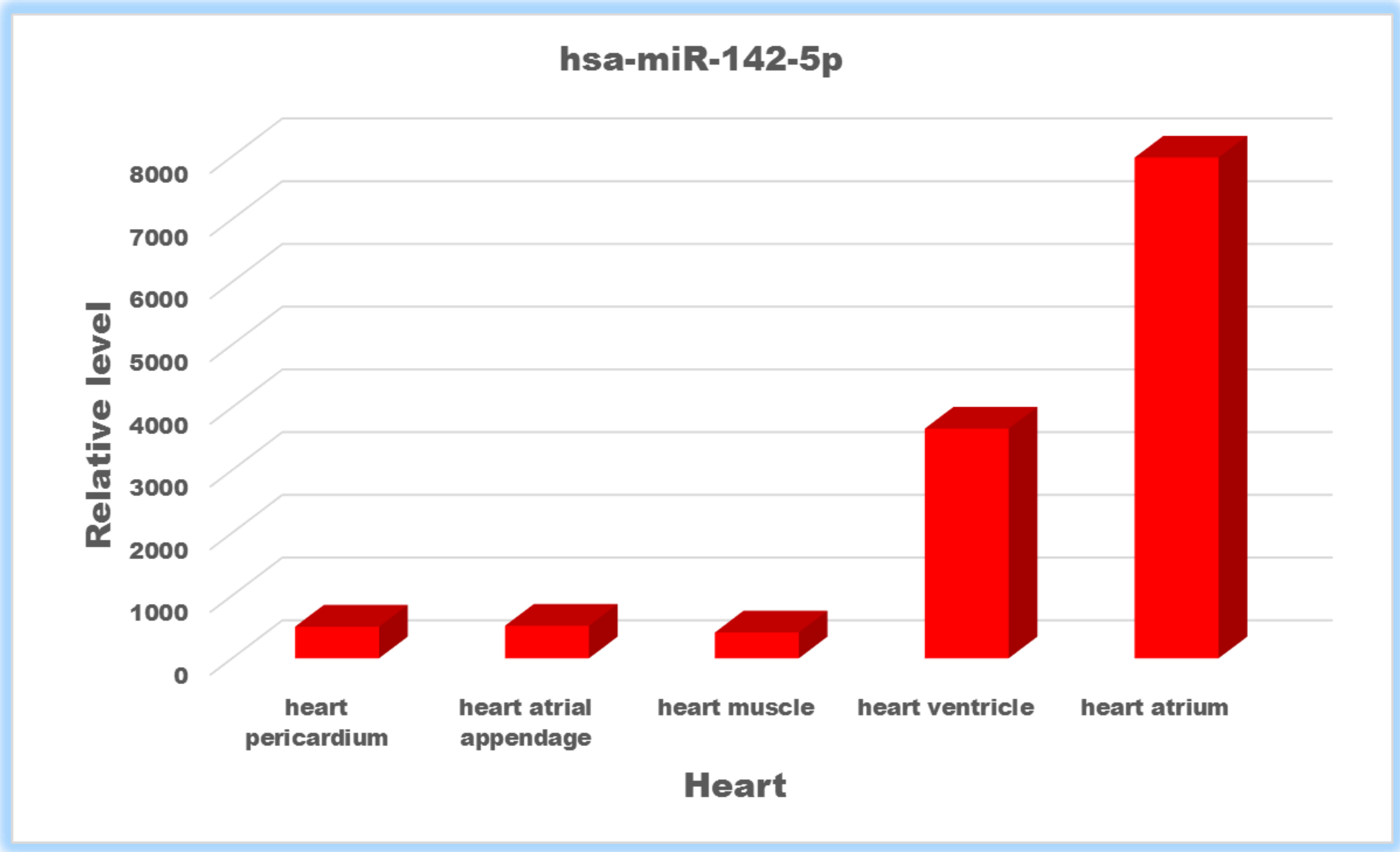

SUPPLE FIG. 6

Profiling expression of the intersection miRNAs in the heart.

A, Expression heatmap of hsa-miR-142-5p, hsa-miR-5590-3p, hsa-miR-579-3p, and hsa-miR-664b-3p in the heart. B, Profiling of hsa-miR-142-5p in all parts of the heart. Among four intersection miRNAs, hsa-miR-142-5p showed high expression in distinct parts of the heart.

SUPPL FIG.7

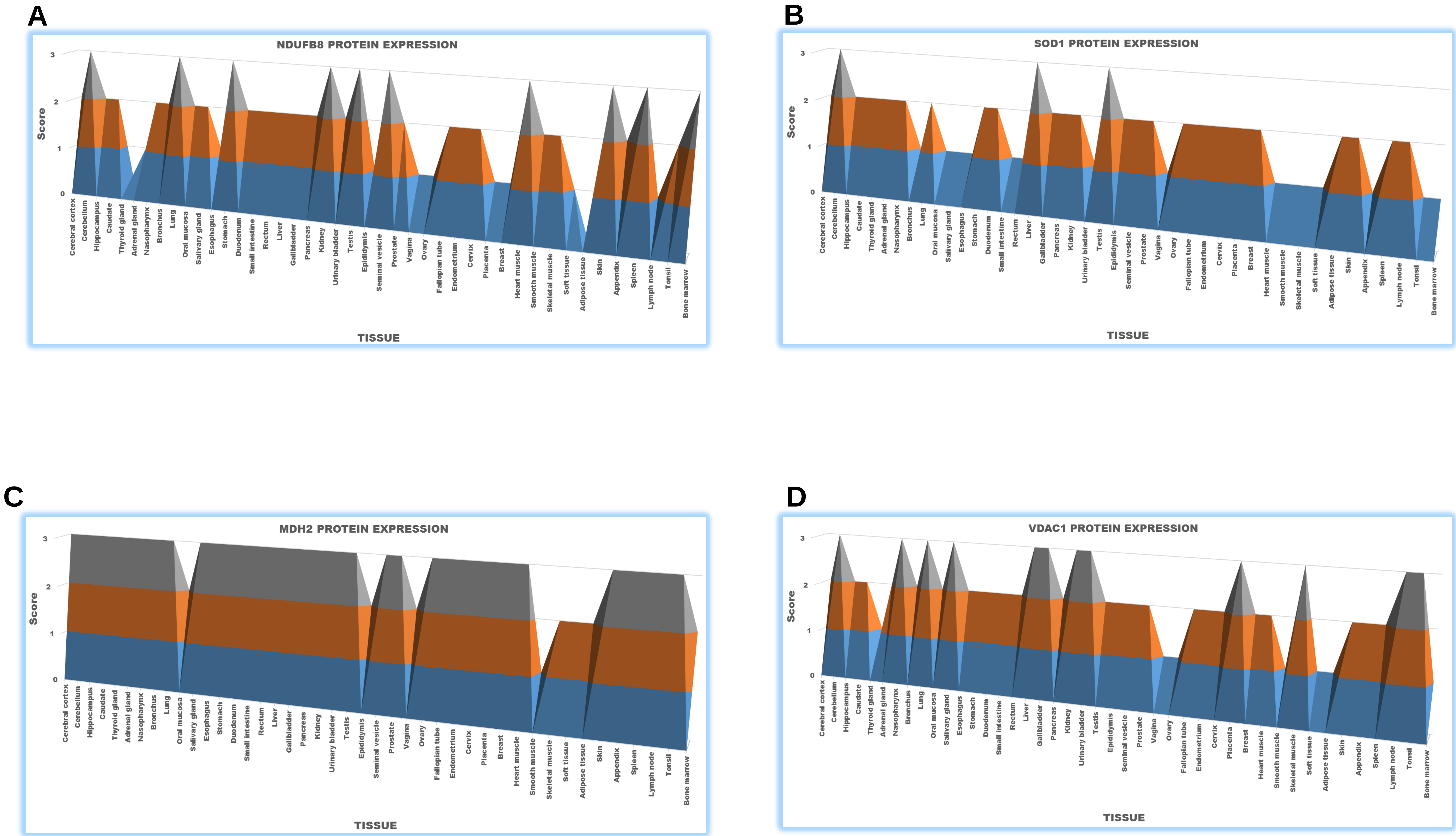
A
NDUFB8 PROTEIN EXPRESSION
Score
0
1
2
3
Cerebral cortex
Cerebellum
Hippocampus
Caudate
Thyroid gland
Adrenal gland
Nasopharynx
Bronchus
Lung
Oral mucosa
Salivary gland
Esophagus
Stomach
Duodenum
Small intestine
Rectum
Liver
Gallbladder
Pancreas
Kidney
Urinary bladder
Testis
Epididymis
Seminal vesicle
Prostate
Vagina
Ovary
Fallopian tube
Endometrium
Cervix
Placenta
Breast
Heart muscle
Smooth muscle
Skeletal muscle
Soft tissue
Adipose tissue
Skin
Appendix
Spleen
Lymph node
Tonsil
Bone marrow
TISSUE
B
SOD1 PROTEIN EXPRESSION
Score
0
1
2
3
Cerebral cortex
Cerebellum
Hippocampus
Caudate
Thyroid gland
Adrenal gland
Nasopharynx
Bronchus
Lung
Oral mucosa
Salivary gland
Esophagus
Stomach
Duodenum
Small intestine
Rectum
Liver
Gallbladder
Pancreas
Kidney
Urinary bladder
Testis
Epididymis
Seminal vesicle
Prostate
Vagina
Ovary
Fallopian tube
Endometrium
Cervix
Placenta
Breast
Heart muscle
Smooth muscle
Skeletal muscle
Soft tissue
Adipose tissue
Skin
Appendix
Spleen
Lymph node
Tonsil
Bone marrow
TISSUE
C
MDH2 PROTEIN EXPRESSION
Score
0
1
2
3
Cerebral cortex
Cerebellum
Hippocampus
Caudate
Thyroid gland
Adrenal gland
Nasopharynx
Bronchus
Lung
Oral mucosa
Salivary gland
Esophagus
Stomach
Duodenum
Small intestine
Rectum
Liver
Gallbladder
Pancreas
Kidney
Urinary bladder
Testis
Epididymis
Seminal vesicle
Prostate
Vagina
Ovary
Fallopian tube
Endometrium
Cervix
Placenta
Breast
Heart muscle
Smooth muscle
Skeletal muscle
Soft tissue
Adipose tissue
Skin
Appendix
Spleen
Lymph node
Tonsil
Bone marrow
TISSUE
D
VDAC1 PROTEIN EXPRESSION
Score
0
1
2
3
Cerebral cortex
Cerebellum
Hippocampus
Caudate
Thyroid gland
Adrenal gland
Nasopharynx
Bronchus
Lung
Oral mucosa
Salivary gland
Esophagus
Stomach
Duodenum
Small intestine
Rectum
Liver
Gallbladder
Pancreas
Kidney
Urinary bladder
Testis
Epididymis
Seminal vesicle
Prostate
Vagina
Ovary
Fallopian tube
Endometrium
Cervix
Placenta
Breast
Heart muscle
Smooth muscle
Skeletal muscle
Soft tissue
Adipose tissue
Skin
Appendix
Spleen
Lymph node
Tonsil
Bone marrow
TISSUE

SUPPLE FIG. 7

Tissue expression profiling of the intersection genes between coRNA and interRNA genes

A, Tissue expressions of NDUFB8. B, Tissue expressions of SOD1. C, Tissue expressions of MDH2. D, Tissue expressions of VDAC1.

SUPPL FIG.8

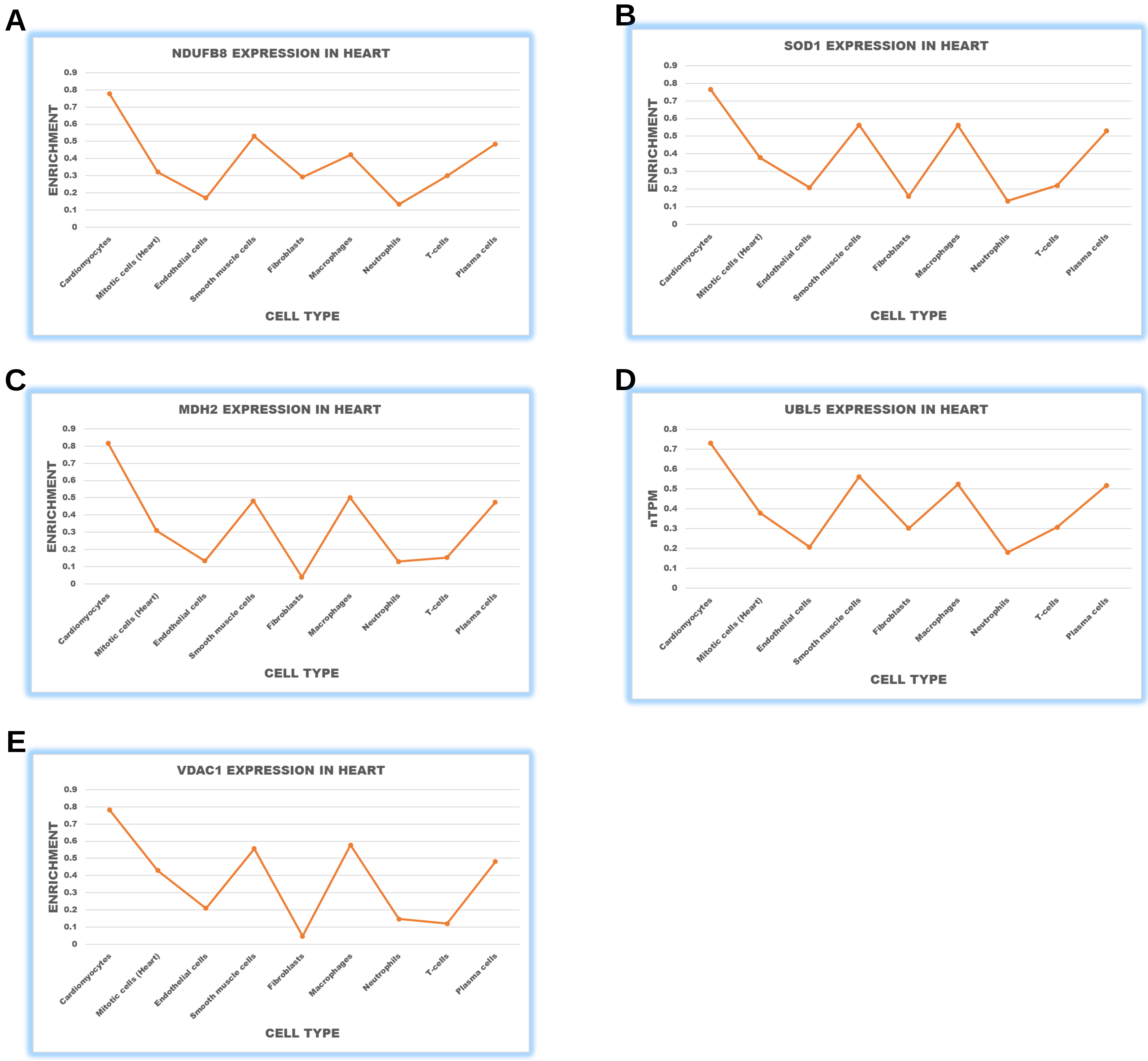
A
NDUFB8 EXPRESSION IN HEART
ENRICHMENT
CELL TYPE
B
SOD1 EXPRESSION IN HEART
ENRICHMENT
CELL TYPE
C
MDH2 EXPRESSION IN HEART
ENRICHMENT
CELL TYPE
D
UBL5 EXPRESSION IN HEART
nTPM
CELL TYPE
E
VDAC1 EXPRESSION IN HEART
ENRICHMENT
CELL TYPE
Cardiomyocytes
Mitotic cells (Heart)
Endothelial cells
Smooth muscle cells
Fibroblasts
Macrophages
Neutrophils
T-cells
Plasma cells

SUPPLE FIG. 8

Cell expression profiling of the intersection genes between coRNA and interRNA genes in distinct heart cell types.

A, Cell expressions of NDUFB8. B, Cell expressions of SOD1. C, Cell expressions of MDH2. D, Cell expressions of UBL5. E, Cell expressions of VDAC1.

SUPPL FIG.8

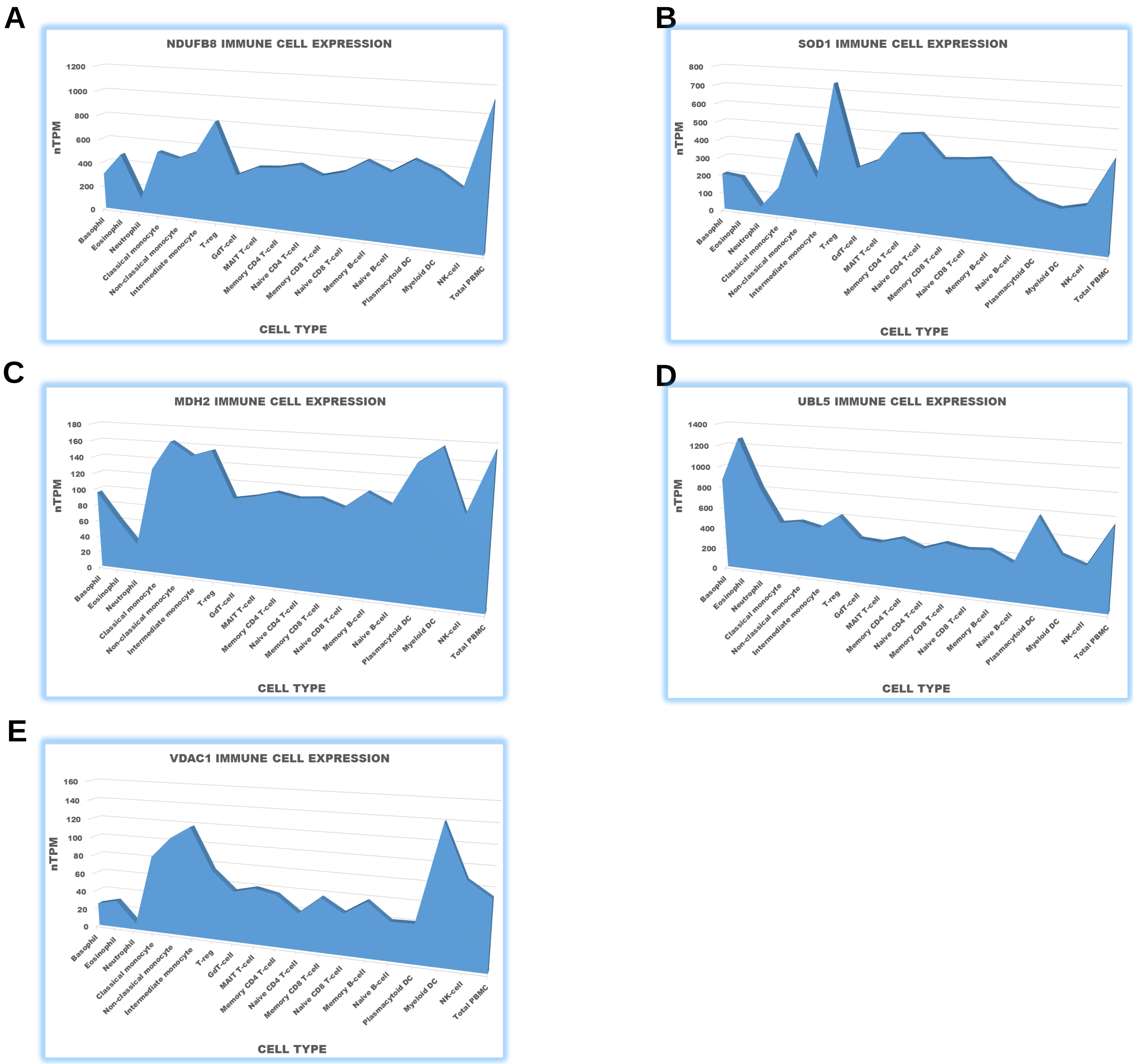
A
NDUFB8 IMMUNE CELL EXPRESSION
nTPM
0
200
400
600
800
1000
1200
Basophil
Eosinophil
Neutrophil
Classical monocyte
Non-classical monocyte
Intermediate monocyte
T-reg
GdT-cell
MAIT T-cell
Memory CD4 T-cell
Naive CD4 T-cell
Memory CD8 T-cell
Naive CD8 T-cell
Memory B-cell
Naive B-cell
Plasmacytoid DC
Myeloid DC
NK-cell
Total PBMC
CELL TYPE
B
SOD1 IMMUNE CELL EXPRESSION
nTPM
0
100
200
300
400
500
600
700
800
CELL TYPE
C
MDH2 IMMUNE CELL EXPRESSION
nTPM
0
20
40
60
80
100
120
140
160
180
CELL TYPE
D
UBL5 IMMUNE CELL EXPRESSION
nTPM
0
200
400
600
800
1000
1200
1400
CELL TYPE
E
VDAC1 IMMUNE CELL EXPRESSION
nTPM
0
20
40
60
80
100
120
140
160
CELL TYPE

SUPPLE FIG. 9

Analyzing immune cell-type specificity of the expression of the five intersection genes between coRNA and interRNA genes.

Extensive expressions in different immune cell types were found for the five intersected coding genes. A, NDUFB8 expressions. B, SOD1 expressions. C, MDH2 expressions. D, UBL5 expressions. E, VDAC1 expressions.

SUPPL FIG.10

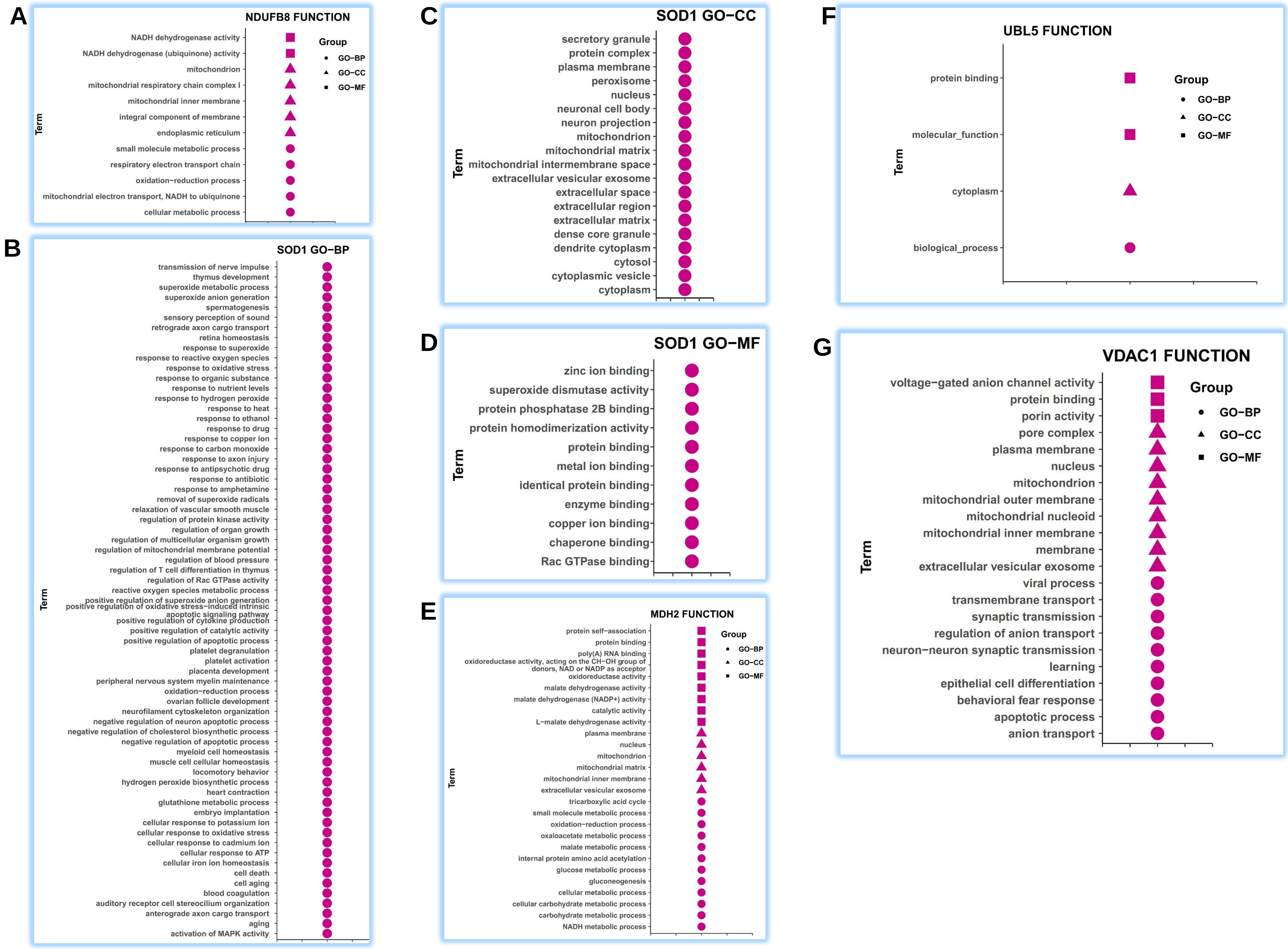
A
NDUFB8 FUNCTION
NADH dehydrogenase activity
NADH dehydrogenase (ubiquinone) activity
mitochondrion
mitochondrial respiratory chain complex I
mitochondrial inner membrane
integral component of membrane
endoplasmic reticulum
small molecule metabolic process
respiratory electron transport chain
oxidation−reduction process
mitochondrial electron transport, NADH to ubiquinone
cellular metabolic process
Term
Group
GO−BP
GO−CC
GO−MF
B
SOD1 GO−BP
transmission of nerve impulse
thymus development
superoxide metabolic process
superoxide anion generation
spermatogenesis
sensory perception of sound
retrograde axon cargo transport
retina homeostasis
response to superoxide
response to reactive oxygen species
response to oxidative stress
response to organic substance
response to nutrient levels
response to hydrogen peroxide
response to heat
response to ethanol
response to drug
response to copper ion
response to carbon monoxide
response to axon injury
response to antipsychotic drug
response to antibiotic
response to amphetamine
removal of superoxide radicals
relaxation of vascular smooth muscle
regulation of protein kinase activity
regulation of organ growth
regulation of multicellular organism growth
regulation of mitochondrial membrane potential
regulation of blood pressure
regulation of T cell differentiation in thymus
regulation of Rac GTPase activity
reactive oxygen species metabolic process
positive regulation of superoxide anion generation
positive regulation of oxidative stress−induced intrinsic apoptotic signaling pathway
positive regulation of cytokine production
positive regulation of catalytic activity
positive regulation of apoptotic process
platelet degranulation
platelet activation
placenta development
peripheral nervous system myelin maintenance
oxidation−reduction process
ovarian follicle development
neurofilament cytoskeleton organization
negative regulation of neuron apoptotic process
negative regulation of cholesterol biosynthetic process
negative regulation of apoptotic process
myeloid cell homeostasis
muscle cell cellular homeostasis
locomotory behavior
hydrogen peroxide biosynthetic process
heart contraction
glutathione metabolic process
embryo implantation
cellular response to potassium ion
cellular response to oxidative stress
cellular response to cadmium ion
cellular response to ATP
cellular iron ion homeostasis
cell death
cell aging
blood coagulation
auditory receptor cell stereocilium organization
anterograde axon cargo transport
aging
activation of MAPK activity
Term
C
SOD1 GO−CC
secretory granule
protein complex
plasma membrane
peroxisome
nucleus
neuronal cell body
neuron projection
mitochondrion
mitochondrial matrix
mitochondrial intermembrane space
extracellular vesicular exosome
extracellular space
extracellular region
extracellular matrix
dense core granule
dendrite cytoplasm
cytosol
cytoplasmic vesicle
cytoplasm
Term
D
SOD1 GO−MF
zinc ion binding
superoxide dismutase activity
protein phosphatase 2B binding
protein homodimerization activity
protein binding
metal ion binding
identical protein binding
enzyme binding
copper ion binding
chaperone binding
Rac GTPase binding
Term
E
MDH2 FUNCTION
protein self−association
protein binding
poly(A) RNA binding
oxidoreductase activity, acting on the CH−OH group of donors, NAD or NADP as acceptor
oxidoreductase activity
malate dehydrogenase activity
malate dehydrogenase (NADP+) activity
catalytic activity
L−malate dehydrogenase activity
plasma membrane
nucleus
mitochondrion
mitochondrial matrix
mitochondrial inner membrane
extracellular vesicular exosome
tricarboxylic acid cycle
small molecule metabolic process
oxidation−reduction process
oxaloacetate metabolic process
malate metabolic process
internal protein amino acid acetylation
glucose metabolic process
gluconeogenesis
cellular metabolic process
cellular carbohydrate metabolic process
carbohydrate metabolic process
NADH metabolic process
Term
Group
GO−BP
GO−CC
GO−MF
F
UBL5 FUNCTION
protein binding
molecular_function
cytoplasm
biological_process
Term
Group
GO−BP
GO−CC
GO−MF
G
VDAC1 FUNCTION
voltage−gated anion channel activity
protein binding
porin activity
pore complex
plasma membrane
nucleus
mitochondrion
mitochondrial outer membrane
mitochondrial nucleoid
mitochondrial inner membrane
membrane
extracellular vesicular exosome
viral process
transmembrane transport
synaptic transmission
regulation of anion transport
neuron−neuron synaptic transmission
learning
epithelial cell differentiation
behavioral fear response
apoptotic process
anion transport
Term
Group
GO−BP
GO−CC
GO−MF

SUPPLE FIG. 10 Functional comments of the five intersection genes between coRNA and inerRNA genes

Annotations of GO were recovered and visualized. A, Functional terms for NDUFB8. B-D, Functional terms for SOD1. E, Functional terms for MDH2. F, Functional terms for UBL5. G, Functional terms for VDAC1. GO, Gene Ontology; BP, biological process; CC, cellular component; MF, molecular function.
